# Extrapolation of Inter Domain Communications and Substrate Binding Cavity of Camel HSP70 1A: A Molecular Modeling and Dynamics Simulation Study

**DOI:** 10.1371/journal.pone.0136630

**Published:** 2015-08-27

**Authors:** Saurabh Gupta, Atmakuri Ramakrishna Rao, Pritish Kumar Varadwaj, Sachinandan De, Trilochan Mohapatra

**Affiliations:** 1 Centre for Agricultural Bioinformatics, ICAR-Indian Agricultural Statistics Research Institute, Pusa, Library Avenue, New Delhi, 110012, India; 2 Department of Bioinformatics, Indian Institute of Information Technology, Allahabad, 210012, India; 3 Animal Biotechnology Centre, ICAR-National Dairy Research Institute, Karnal, 132001, India; 4 ICAR-Central Rice Research Institute, Cuttack, Odisha, 753 006, India; National Institute of Plant Genome Research (NIPGR), INDIA

## Abstract

Heat shock protein 70 (HSP70) is an important chaperone, involved in protein folding, refolding, translocation and complex remodeling reactions under normal as well as stress conditions. However, expression of HSPA1A gene in heat and cold stress conditions associates with other chaperons and perform its function. Experimental structure for Camel HSP70 protein (cHSP70) has not been reported so far. Hence, we constructed 3D models of cHSP70 through multi- template comparative modeling with HSP110 protein of *S*. *cerevisiae* (open state) and with HSP70 protein of *E*. *coli* 70kDa DnaK (close state) and relaxed them for 100 nanoseconds (ns) using all-atom Molecular Dynamics (MD) Simulation. Two stable conformations of cHSP70 with Substrate Binding Domain (SBD) in open and close states were obtained. The collective mode analysis of different transitions of open state to close state and vice versa was examined via Principal Component Analysis (PCA) and Minimum Distance Matrix (MDM). The results provide mechanistic representation of the communication between Nucleotide Binding Domain (NBD) and SBD to identify the role of sub domains in conformational change mechanism, which leads the chaperone cycle of cHSP70. Further, residues present in the chaperon functioning site were also identified through protein-peptide docking. This study provides an overall insight into the inter domain communication mechanism and identification of the chaperon binding cavity, which explains the underlying mechanism involved during heat and cold stress conditions in camel.

## Introduction

Camels (*Camelus dromedarius*) are working animals with tasks varying from human transport to bearing loads and also provide milk, meat and hair. Hence, the farming of camel is an important source of livelihood for farmers of arid regions of Africa, South America and Asia (mainly India, Pakistan, and Afghanistan) [1, 2]. The ability to survive and adapt to thermal stress plays an essential role in identifying the distribution and performance of camel [3, 4]. The adaptive responses come from considerable increment in the expression of genes related to cell protection from abiotic stresses. These genes encode for a family of Heat shock proteins (HSPs) and cytoprotective proteins as well as other molecular chaperones [5]. HSPs are highly conserved across evolutionary line and consist of 2–15% of the total cellular proteins in all living organism [6]. They play an important role in the maintenance of intracellular homeostasis and in preventing the aggregation of stress induced or disease induced cellular proteins by controlling the protein folding process. Besides, they exhibit anti-apoptotic properties and modulate various immune related responses. Mammalian genome encodes for more than 100 different types of HSPs [7]. HSPA type is an important group of HSP and commonly known as HSP70. In humans, HSPA (HSP70) contains at least 13 members, out of which some are highly heat stress inducible and others are constitutively expressed [8]. HSP70 members are found in all the major sub-cellular compartments and play important role to protect cellular proteins against stress. Cellular stresses like elevated temperature, mechanical damage, hypoxia, lowered pH and reactive oxygen species generation may alter protein folding leading to higher HSP70 gene expression [9]. HSP70s are highly conserved proteins and are present in almost all mammalian species. Within the HSPA family (HSP70), there are two genes, namely, HSPA2 and HSPA1L that are originally identified as highly specific to some vital roles including spermatogenesis.

The structural constituents of HSP70 protein consists of two highly conserved functional domains, *viz*., Nucleotide binding domains (NBD) and Substrate binding domain (SBD). The NBD is divided into four or five sub-domains and separated into two lobes by middle ATP binding cleft whereas the SBD, consists of β-sheet rich substrate binding (SBD- β) cavity and α-helical part (SBD-α) (also referred as ‘lid’),which forms a cover on the cavity. All functions of HSP70 rely on the fleeting interaction between SBD-β with a small segment of polypeptide as a substrate. This interaction is regulated by the nucleotide status of NBD. Binding of ATP with NBD (ATP-bound state) increases the rate of association and dissociation of substrates (substrate rate exchange), while substrate affinity remains low. Hydrolysis of ATP changes the nucleotide status of NBD known as ADP-bound state. In this state, affinity for substrate is high while substrate exchange rates are low [10, 11, 12]. Moreover, interaction of hydrophobic peptide segment of other proteins with SBD causes conformational change within the SBD that imitate ATP hydrolysis in proportion to its binding affinity. This shows a clear inter-domain communication mechanism for regulation of HSP70 functions [13].

It has been reported that the peripheral blood mononuclear cells (PBMCs) of cattle and buffalo express more HSP70 in response to heat shock at 42°C in vitro [14]. Five HSP70 member genes, v*iz*., HSPA8, HSPA6, HSPA1A, HSPA1L and HSPA2 were identified and quantified in Indian heat- and cold-adapted goats (*Capra hircus*). During summer, the expression of HSPA8, HSPA6 and HSPA1A was high while the expression level of HSPA1L and HSPA2 was low, whereas the expression of HSPA1A and HSPA8 was high during winter in both heat- and cold-adapted goats but low in case of other HSPs. Besides, the expression of HSPA1A gene was found higher in both heat and cold stress conditions [15]. Thus, study of HSPA1A or homologues genes which express HSP701A protein become relevant for structural chaperon functional activity in camel. In this paper, an attempt has been made to identify inter-domain communication between SBD and NDB of cHSP70 through all atom molecular modeling and dynamics simulations and identify chaperon cavity of SBD of cHSP70 through protein-peptide docking.

## Material and Methods

### Identification and Evolutionary analysis of cHSP70

The prime concern of the present study was to identify three-dimensional (3D) structures and functional features of cHSP70 protein expressed by HSPA1A gene [16]. For this, we have collected all available full length HSP70 1A protein sequences from UniProt, which are translated from HSPA1A and its homologue HSPA1B (S1 Table). A manual screening was performed by excluding partial, putative, uncharacterized and hypothetical sequences during sequence selection. A total of 33 protein sequences of HSP70 1A from species like Human (*Homo sapience*), Mouse (*Mus musculus*), Cow (*Bos taurus* / *Bos indicus*), Goat (*Capra hircus*), Sheep (*Ovis aries*), Buffalo (*Bubalus bubalis*), Gaur or Indian Bison (*Bos gaurus*), Tape worm (*Echinococcus multilocularis*), Hamster (*Cricetulus griseus*), Asian swamp *eel* (*Monopterus albus*) and Sumatran orangutan (*Pongo abelii*) were found. To identify the homology of the HSPA1A/1AB gene in different species, the Multiple Sequence Alignment (MSA) was performed using CLC-Genomics workbench v7.0.5 (CLC Bio, Aarhus, Denmark). Subsequently, phylogenetic analysis was done using Neighbor-Joining method of CLC-Genomics Workbench for identification of evolutionary relationship between the proteins of camel and other *species*. Moreover, we have performed MSA among closely related 17 HSP70 sequences of Cow, Buffalo, Goat and Sheep to identify similarities and dissimilarities between the protein sequences across species.

### Generation of 3D-models

The position of SBD-α relative to SBD-β defines two main states of HSP70 protein: either the lid is open where the peptide can access the hydrophobic pocket within SBD-β named as ‘open state’—*Initial open model (IOM) of camel HSP70* or the lid is closed where the peptide is ensnared in the pocket known as ‘closed state’—*Initial closed model (ICM) of camel* HSP70 [12]. Initially, protein models of both states of cHSP70 were generated through multi-template comparative modeling. Domain Enhanced Lookup Time Accelerated BALST (DELTA-BLAST) search was then performed using cHSP70 sequence as query against Protein Data Bank (PDB). All best screened homologous protein 3D structures and their experimental information was analyzed [17]. The 3D structures of IOM-cHSP70 and ICM-cHSP70 were generated using MODELLER (v9.12) [18]. The loop modeling and refinement was performed to maximize the model accuracy using Modloop server [19]. After loop refinement, a comparative analysis was made between initial and refined models by means of several validation measures. Structure Analysis and Verification Server v4.0 (SAVES) was used to evaluate overall validation measures of 3D structures. The energy minimization of refined IOM-cHSP70 and ICM-cHSP70 model was performed using AMBER ff12SB force field using steepest descent algorithm for 1000 runs using Chimera [20]. The most stable models were submitted in ProFunc server for the identification of biochemical functions and prediction of domains (S1 Fig)[21]. Finally, the stable models were submitted for all atoms MD simulation.

### Molecular Dynamics Simulation

All atoms MD simulations for IOM-cHSP70 and ICM-cHSP70 have been performed using simple point charge (SPC) water model using GROMACS v4.6.2 [22, 23]. Time step used in both simulations was 2 Ferro seconds (fs) and neighbor search list was updated in every 10fs using grid method. In every 2 Pico second (ps) the coordinates of all atoms were updated in the cubic simulation box. The required Sodium ions (Na^+^) were added for neutralizing the acidic environment of the box. The Canonical ensemble (NVT) and Isothermal-isobaric ensemble (NPT) were used with normal temperature (300K) and pressure (tau p = 1ps). Particle Mesh Ewald (PME) algorithm was used for electrostatic term computation. Non-columbic potentials within 1nm radius were controlled by cut-off algorithms. The refined models of IOM-cHSP70 and ICM-cHSP70 were subjected to molecular dynamics simulation using GROMOS53a6 force field [24]. Further, IOM-cHSP70 and ICM-cHSP70 were solvated in cubic box with 151352 and 144790 SPC water molecules respectively [25]. The water molecule was having minimum distance of 1.0 nm between the solute and each face of the box. The periodic boundary of 10nm long from each side of the box was used. Charges of both systems have been neutralized by adding 9 Na^+^ counter ions. The system energy was optimized using “steepest descent minimization” algorithm followed by “conjugate gradient” algorithm. The production run period was set for 100ns and basic trajectory analysis was performed using various GROMACS inbuilt programs. In addition, graphs and figures were generated using Grace and Chimera [20].

### Principal component analysis and Minimum distance matrix analysis of MD trajectory

Protein's function is often linked with its conformational dynamics/changes and it is not always straight forward to extract the functionally relevant motions from a simulated MD trajectory. However, Principal Component Analysis (PCA) takes the trajectory of a molecular dynamics simulation and extracts the dominant modes in the motion of the molecule. Initially, a covariance matrix is generated from the trajectory of N atoms of a protein and then a diagonalization of the covariance matrix was done under PCA. The columns of this transformed matrix are known as eigenvectors or principal components, where each of the components is associated with an eigenvalue representing the energy contribution of that particular component to the motion. The principal components provide vector description of each component of the motion, by indicating the direction of the motion. Any projection of the trajectory on a particular eigenvector highlights the time-dependent motion of that component in a particular vibrational mode and the time-average of the projection shows the contribution of components of the atomic vibrations to the mode of concerted motion [26]. Thus, PCA was used in MD simulations to analyze the motions contributing to the overall dynamics of cHSP70 protein. In this procedure, a total of (3N-6) number of modes of possible internal fluctuations exists for a molecule with N number of atoms. Here, value 6 indicates the degrees of freedom essential to describe the external rotation and translation of the system [27]. The *g_covar* of GROMACS was used to diagonalize the covariance matrix *σ*_*ij*_ = (*∂α*_*i*_ *∂α*_*j*_), from their trajectory-averaged values, where *∂α*_*i*_ = *α*_*i*_ *−*〈*α*_*i*_〉,*∂α*_*j*_ = *α*_*j*_ *-*〈*α*_*j*_〉,*α*_*i*_ = {*x*_*i*_,*y*_*i*_,*z*_*i*_},*α*_*j*_ = {*x*_*j*_,*y*_*j*_,*z*_*j*_}. The symbol “〈…〉" indicates the trajectory average and { , , } indicates the Cartesian coordinates of an atom. Further, *g_covar* decomposes the configuration point ***r*** ^3N^(t) = (***x***_***1***_(***t***),***y***_***1***_(***t***),…,***z***_***n***_(***t***))^***T***^ as

$$\sum_{i=1}^{3N}\left[r^{3N}\left(t\right).m_{i}\right]m_{i}=\sum_{i=1}^{3N}p_{i}\left(t\right)m_{i}$$ (1)

where ***m***_***i***_ is the orthogonal eigenvector of the covariance matrix with corresponding eigenvalue denoted by $\lambda_{i}^{2}$. In the rotated Cartesian coordinate basis defined by ***m***_***i***_ **(*i = 1*, *2*, *…*, *3N*),** the largest eigenvalue captures the first largest fraction of root-mean-square fluctuation (RMSF), the second largest captures the next largest fraction of RMSF and so on. Then these fractions are arranged in a descending order to the protein’s fluctuation in small set modes. ***p***_***i***_(***t***) is the projection on the configuration ***r*** ^3N^ versus projection on the deviationfrom the trajectory average, *i*.*e*., ***δr*** ^***3N***^(***t***) = ***r*** ^***3N***^(***t***)-〈***r*** ^***3N***^(***t***)〉 from the trajectory average. The contribution of i^th^ mode fluctuation of atom j is obtained from

$$\left|m_{i}^{j}\right|=\sqrt{{\left(m_{i}^{jx}\right)}^{2}+{\left(m_{i}^{jy}\right)}^{2}+{\left(m_{i}^{jz}\right)}^{2}}\equiv Component_{i}^{j}$$ (2)

where $m_{i}^{j}\;=\;\left\{m_{i}^{jx},m_{i}^{jy},m_{i}^{jz}\right\}$ is referred as *component vectors* of j^th^ atom for the i^th^ mode. The total RMSF^**2**^ is decomposed as

$${\text{RMSF}}^{2}=\Sigma_{i}\lambda_{i}^{2}=\Sigma_{i}\lambda_{i}^{2}m_{i}.m_{i}=\Sigma_{i}\Sigma_{j}\lambda_{i}^{2}\left(m_{i}^{jx}m_{i}^{jx}+m_{i}^{jy}m_{i}^{jy}+m_{i}^{jz}m_{i}^{jz}\right)=\Sigma_{i}\Sigma_{j}{\left(\lambda_{i}Component_{i}^{j}\right)}^{2}\equiv \Sigma_{i}\Sigma_{j}{\left(R-Component_{i}^{j}\right)}^{2}$$ (3)

where the product ${\;\lambda }_{i}{Component}_{i}^{j}\equiv {R-Component}_{i}^{j}$ is the contribution of atom *j* in mode *i* to the total fluctuation of protein [28]. Whereas, *g_anaeig* of GROMACS was used to analyze and plot the principal components. Moreover, harmonic motions of residues describe the collective mode of transitions among different states of protein (open and close state). The evaluation of contribution of these modes was calculated by the Involvement (I_k_) and Cumulative Involvement coefficients (CI_k_), where k is the collective mode for the projection of the atomic displacements within k^th^ mode. In addition, the dominant motions of backbone atoms of ICM-HSP70 and IOM-HSP70 were also analyzed for every successive 10ns time intervals [29, 30].

The Minimum Distance Matrix (MDM) is an N×N symmetric matrix (N is the number of residues) which shows the smallest distance between the atoms of pairs of residues. MDM was calculated for both IOM-cHSP70 and ICM-cHSP70 trajectories at 10ns intervals to investigate the expected change in the patterns or location of alpha-helix, beta sheet and other structural elements by a contour map. The *g_mdmat* of GROMACS was used to calculate the MDM and the *g_mdmat* with–*frames* option was used to store the distance matrices to see the structural differences of protein as a function of time. An averaged matrix over the whole trajectory was used to identify pattern and locational changes in the secondary and tertiary structures of the protein. *Xpm2ps* of GROMACS was further used to generate the different maps.

### Peptide modeling and minimization

Recently, Xu, et al. (2012) used seven different peptides and identified the efficacy of peptide binding in the hydrophobic SBD-β of HSP110 for determination of its distinct chaperone activity [31]. Hence, we have considered all the seven reported peptides of Xu, et al. (Table 1) as substrates to identify the common substrate binding residues in the SBD-β domain of cHSP70. Prior to substrate docking, peptides were modeled by the de-novo peptide structure prediction servers: PEP-FOLD and PEPSTR [32, 33]. The PEP-FOLD server, in general model the peptides of 9–36 amino acids long sequence in aqueous solution using a Hidden Markov Model. A total of six peptides structures, except NR which has 7 residues, were generated by the server after execution of a series of 50 simulation runs. This has led to the identification of most representative conformation in terms of energy and population. Since, NR peptide does not fulfill the condition of minimum residue requirement of structure prediction in PEP-FOLD server the structure of this peptide was generated by PEPSTR server that is capable to build peptides between 7–25 residues in hydrophilic environment. In order to get stable structure, the energy minimization was performed by above said severs. The predicted peptide models were analyzed and the lowest energy model was selected for protein-peptide docking.

**Table 1:** List of HSP70 substrate binding peptides reported by Xu.et al. in 2012.

S. No.	Peptide Name	Sequence	Sequence length
1	TRP2	SVYDFFVWL	9
2	p53	LDGEYFTLQIRGRER	15
3	NR	NRLLLTG	7
4	p12	LQSRLLLSAPRR	12
5	TRP2_F5L/F6L	SVYDLLVWL	9
6	TRP2_W8L	SVYDFFVLL	9
7	TRP2_181	VYDFFVWLHYY	11

### Docking and refinement of peptides in SBD-β

The substrate binding residues of SBD-β of cHSP70 were identified by performing structural alignment of modeled cHSP70 using Dali server[34]. The alignment reveals probable substrate binding residues in cHSP70 are Glu 404, Ala 406, Ala 429, Tyr 431, Gln 435, Leu 439 and Gln 441. The affinity of binding residues with respect to modeled peptides was initially confirmed by performing the protein-peptide docking using ZDOCK server [35]. Docking was performed in two ways (i) Blind Docking, where the peptides are free to dock any cavity of the SBD-β (ii) Site specific Docking, where peptides are docked in the assigned protein binding site. Complex docking structures of SBD-β and peptides, produced by both of the aforementioned methods, were analyzed on the basis of their binding energy and Z-Score [36]. Subsequently, the best selected complex for each peptide was submitted to Rosetta FlexPepDock server for the refinement [37]. The server yields 1000 decoys of protein-peptide complex. Finally, seven best complexes, one each from seven protein-peptide complexes were selected for SBD-β-peptide interactions analysis by LIGPLOT [38].

## Results and Discussion

### Evolutionary sequence analysis of cHSP70

The phylogenetic analysis of the selected HSP70 protein sequences is given in S2 Fig. The phylogenetic tree reveals that cHSP70 1A protein, expressed by HSPA1A gene, show less divergence with HSP70 of Cow, Buffalo, Goat, Sheep and human whereas, more divergence with HSP70 of Mouse, Rat, Hamster, Tap worm, Sumatran orangutan and Asian swamp eel. This evolutionary analysis confirms that the selected cHSP70 protein may function in a similar way as that of HSP70 of Goat, Buffalo, Cow and Sheep species. The DELTA BLAST of cHSP70 has identified different templates from PDB of which the best identified templates were 3C7N Chain B, 1YUW Chain A, and 2V7Z Chain A [39, 40, 41]. Though the 3D information of these templates does not fully cover the whole sequence of cHSP70, an amount of 86% (554 out of 641 residues) of the sequence has aligned with the Chain B of 3C7N and chain A of 1YUW separately. Also, 84% (544 out of 643 residues) of cHSP70 sequence are found to be aligned with chain A of 2V7Z. On the other hand, few other templates shared low identity but high coverage with cHSP70. Among them, Chain A of 2KHO [42] showed 95% coverage (613 out of 641 residues) and shared 49% (304/623) identity with cHSP70. Whereas, the Chain A of 4JN4 template showed 94% coverage (608 residues out of 641) and shared 48% (296/615) identity with cHSP70. Thus Hsp110 homologue of *S*. *cerevisiae* (PDB ID: 3C7N) and HSP70 of (DnaK) chaperone32 of *E*. *coli* (PDB ID: 2KHO) were used as templates for modeling of IOM-cHsp70 and ICM-cHsp70 respectively.

### Modeling and structural analysis of IOM-cHsp70 and ICM-cHsp70

**Fig 1:**
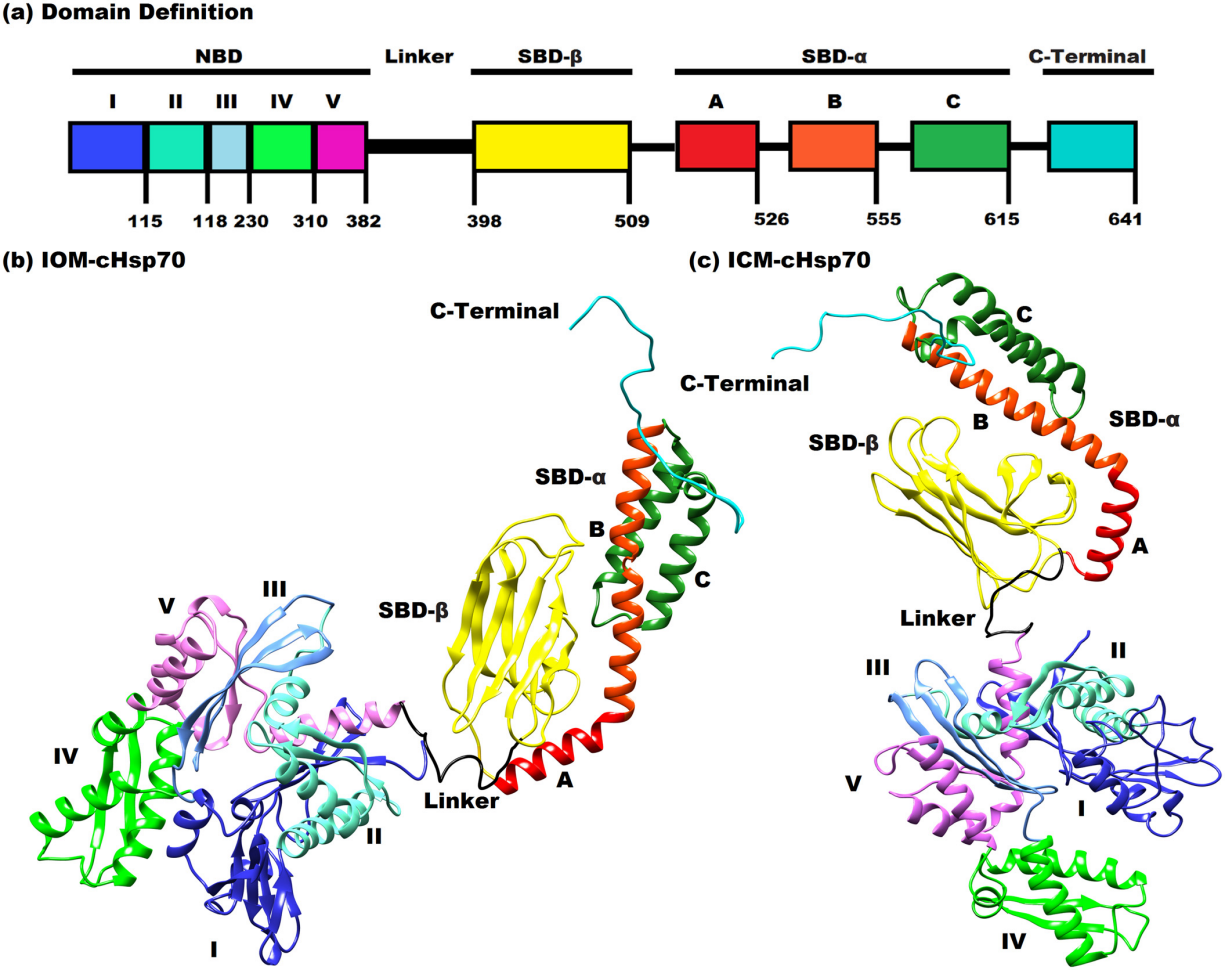
(a) Definition of secondary structure of cHSP70. The color code is as follows: NBD (residues 1–382): sub-domain I (residues 1–115; blue), sub-domain II (residues 116–188; light-cyan), sub-domain III (residues 189–230; cornflower blue); sub-domain IV (residues 231–310; green); sub-domain V (residues 311–382; light-magenta), SBD (399–615) is shaped by a *β* sandwich (SBD-β) (residues 399–509 in yellow) and a helix bundle (SBD-α) (residues 510–615; red), Linker (residues 383–398; black) a short peptide connecting NBD & SBD and C-Terminal (residues 616–641; cyan) is relatively an unconstructed chain. (b) Solid ribbon diagram of IOM-cHSP70 built by homology from HSP110 of *Saccharomyces cerevisiae* (PDB ID 3C7N Chain A). (c) Solid ribbon diagram of ICM-cHSP70 build by multi-template comparative modeling from *Escherichia coli* of DnaK structure (PDB ID: 2KHO). The color code for (b) and (c) figures is similar to (a). The figure was prepared with chimera [http://www.cgl.ucsf.edu/chimera].

The IOM-cHsp70 and ICM-cHsp70 models were built and depicted in Fig 1b and 1c respectively. The open state of IOM-cHsp70 structure was built by the multi-template comparative modeling using the structure of Hsp110 of *S*. *cerevisiae*, whereas the close state of ICM-cHSP70 was generated by using close state structure of DnaK of *E*. *coli*. The domain-wise description of cHSP70 is given in Fig 1a. The ranges of residues in different domains are NBD [1–382], Linker [383–397], SBD-β [398–509] SBD-α [510–614] and C-Terminal [615–641]. The main significant structural difference between HSP110 and HSP70 is the bending of A and B helices in the SBD-α sub domain. Hence, the modeled IOM-cHSP70 is having a long helix (A+B) while ICM-cHSP70 is having a ‘kink’ between A and B helices (Fig 1c). Ramachandran Plot statistics (Table 2) and other validation parametric values of IOM-cHSP70 and ICM-cHSP70 models revealed that only 0.7% and 0.2% residues were present in disallowed region respectively. These residues are Ala60, Asp97, Lys190, Leu391, Asp443, Asn505, Ser544 and Lys559 of ICM-cHSP70 and His23, Asp292, Asn355, Leu394, Leu399 and Glu588 of IOM-cHSP70. Through loop modeling and refinement, these residues were remodeled. Refined structures of both models have gained improvement over initial models and can be seen from Ramachandran Plot statistics, ERRAT scores and VERIFY3D scores (Table 2). The chosen scores have been previously used in studying mechanisms of inhibitors and functional analysis [43, 44]. IOM-cHSP70 consists of 8 sheets, 1beta alpha beta unit, 12 beta hairpins, 10 beta bulges, 28 strands, 22 helices, 26 helix-helix interactions, 55 beta turns and 6 gamma turns while ICM-cHSP70 consist of 7 sheets, 1beta-alpha-beta unit, 11 beta hairpins, 8 beta bulges, 24 strands, 18 helices, 15 helix-helix interactions, 50 beta turns and 17 gamma turns. The differences in the secondary structural elements of IOM-cHSP70 and ICM-cHSP70 models are due to the differences in states (open & close states).

**Table 2:** Protein validation statistics for IOM-cHsp70 and ICM-cHsp70 models after a series of modeling and loop refinements.

Ramachandran Plot statistics PROCHECK Score	IOM-cHsp70 (Modeled)	ICM-cHsp70 (Modeled)	IOM-cHsp70 (Refined)	ICM-cHsp70 (Refined)
% Amino acid in most favored regions	90.6%	91.6%	92.2%	92.5%
% Amino acid in additional allowed regions	8.7%	7.1%	7.3%	7.1%
%Amino acids in generously allowed regions	0.5%	0.9%	0.5%	0.4%
% Amino acids in disallowed regions	0.7%	0.2%	0.0%	0.0%
**ERRAT score**	81.952	71.617	82.311	75.556
**VERIFY 3D score**	0.80	0.88	0.87	0.90

### Relaxation of cHSP70 structure by MD Simulation

#### Open state model

**Fig 2:**
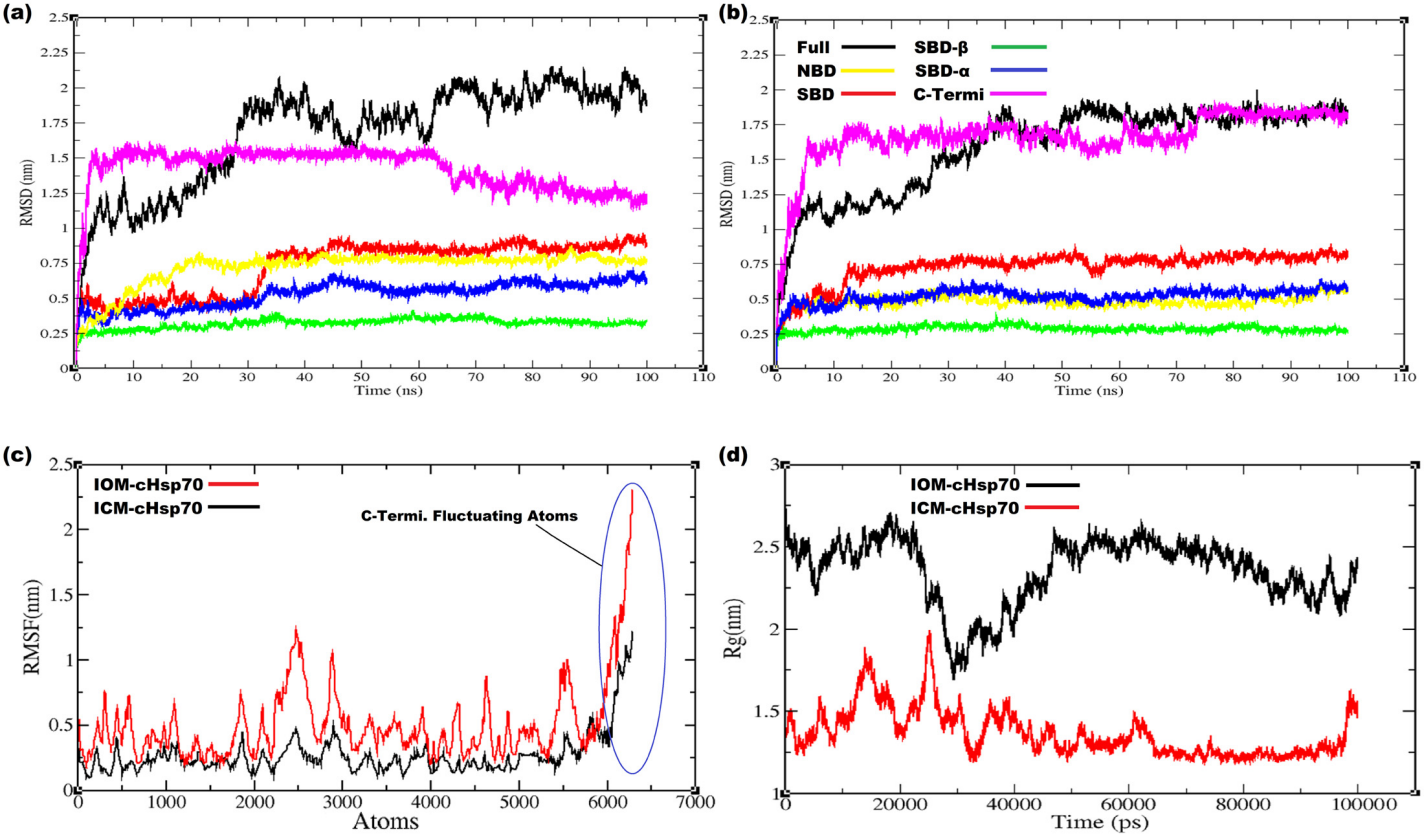
(a) The backbone RMSD for Full structure of IOM-cHSP70 having NBD, SBD, SBD-β, SBD-α and C-Terminal computed with respect to simulation time (b) The backbone RMSD for Full structure of ICM-cHSP70 having NBD, SBD, SBD-β, SBD-α and C-Terminal computed with respect to simulation time(c) RMSF plots of ICM-cHSP70 and IOM-cHSP70 showing atomic fluctuations in (nm) with respect to each atom of the protein. (d) The Radius of gyration (R_g_) plot for both proteins is represented by different color scheme with respect to simulation time.

**Fig 3:**
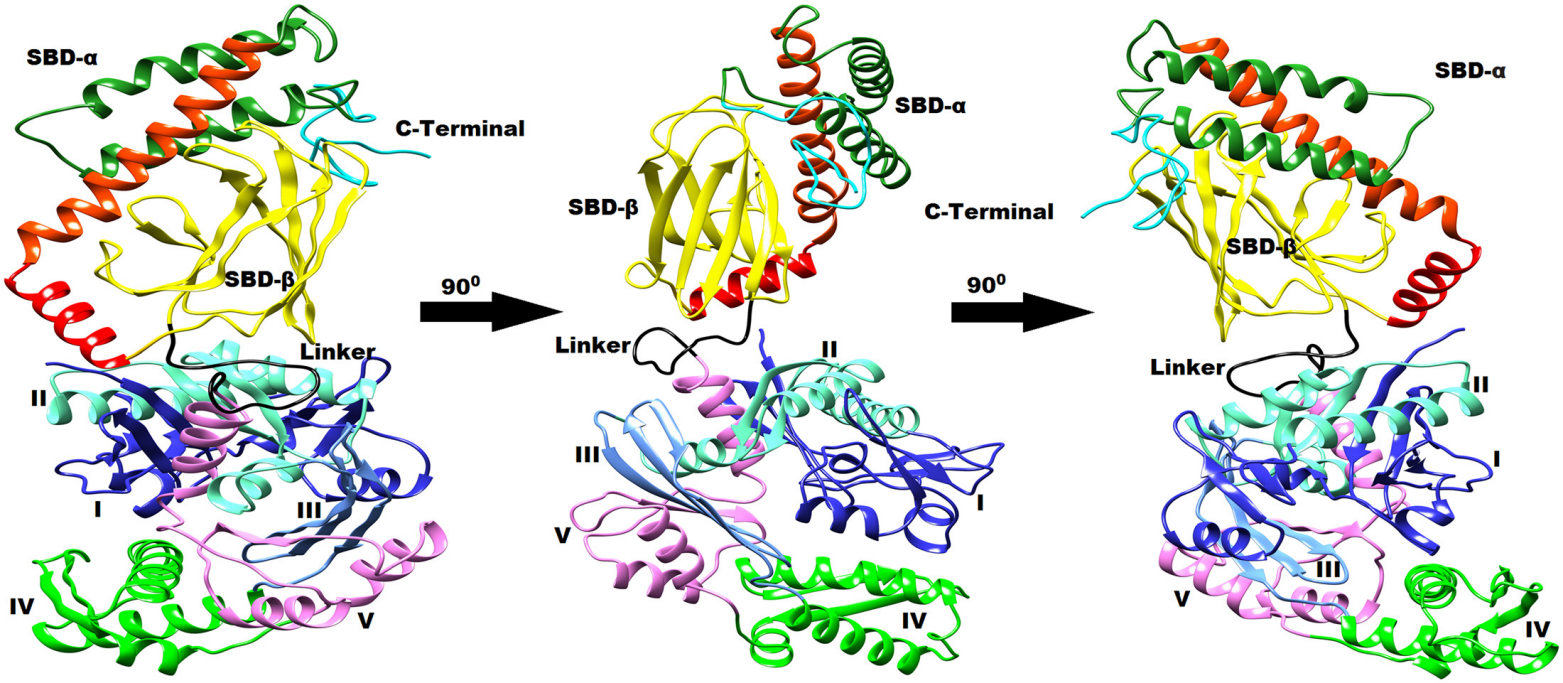
Typical average 3D structure represented in cartoon diagram of open state of cHSP70 rotated by 90° after relaxation through MD simulation.

IOM-cHSP70 was relaxed by all atoms MD simulation at 300 K temperature and 1 bar pressure of system. The stability and structural changes in simulated model were monitored by computing backbone Root Mean Square Deviation (RMSD) with respect to the initial model (for the full structure as well as NDB, SBD, SBD-β, SBD-α and C-terminal domains separately) as a function of time (Fig 2a). The structure of NBD converged to a stable conformation after 22.57 ns, which is evident from the convergence of RMSD, whereas the structure of SBD reached equilibrium after 30.65 ns. Later on a sharp change occurred in structural conformations of SBD during 30.65 ns-39.20 ns. Subsequently, the structure got converged and found to be stable with respect to the initial model. Moreover, the behavior of SBD-β, SBD-α subunits during simulation were found more stable than other units of the structure. Structure of SBD-β unit started converging to a stable conformation after 34.95 ns and found that the unit of RMSD lies between 0.3–0.4nm. RMSD with respect to the initial structure of SBD-α subunit was constantly increased up to 45.10 ns and then tried to stabilize (Fig 2a). Whereas, RMSD (1.58 nm at 3.5 ns) is high in C-Terminal as compared to the RMSD in SBD and NBD. This pattern of RMSD continued up to 63.6 ns and then tried to converge to a stable conformation (Fig 2a). A relatively higher RMSD (2.0 nm, at 35 ns) was observed for whole structure of IOM-cHSP70 with respect to its initial model. This may be due to (i) the restructuring of the NDB, SBD and C-Terminal and (ii) the reorientation of the SBD-β, SBD-α relative to the NBD. A similar RMSD calculation was performed by Nicolai, et al.(2012) in human for open state of HSP70 [45]. Besides the RMSD analysis, the examination of Root Mean Square Fluctuations (RMSF) of atoms of IOM-cHSP70 (Fig 2c) showed two helices (H7 and H8) and one beta hairpin of domain of V of NBD, Linker, Helix C of SBD-α and the C-Terminal (Range of atoms 2400–3000 for NBD, 5400–5600 for SBD-α and 6092–6272 for C-Terminal) were having huge fluctuations, which reflects a reorientation of the structures. The major structural changes took place at about 35 ns (shown as a sharp increase in RMSD at 35 ns for SBD and for full structure shown in Fig 2a) due to (i) breaking of the initial fused helices “A and B” of the SBD-α into separate helices and (ii) binding of the SBD-α on NBD. Later on the RMSD of IOM-cHSP70 has shown more deviation during 35 and 90 ns (Fig 2a). Finally, this behavior of deviation is minimized during 90–100 ns course of simulation and got stable at the end of the MD simulation. In addition, an average structure of IOM-cHSP70, after attaining stability, is given in Fig 3. Longer simulations were not performed due to (i) as they do not significantly change the conformation of HSP70 even after 100 ns in case of human (ii) Limitation computational power.

#### Close state model

**Fig 4:**
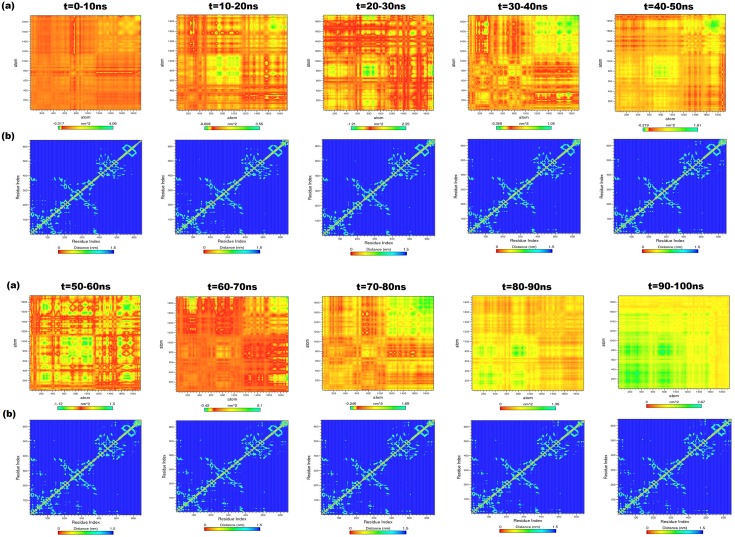
Typical average 3D structure represented in cartoon diagram of close state of cHSP70 rotated by 90° after relaxation through MD simulation.

Trajectory analysis of ICM-cHSP70 through multi-template comparative modeling is performed using different GROMACS trajectory analysis programs. The backbone deviation of structure is measured by calculating RMSD with respect to time. Initially, the structure of ICM-cHSP70 evolved till 40 ns and then reached convergence state after 50 ns (Fig 2b). However, the SBD reached convergence after 13 ns and structure remained with minor backbone fluctuations till end. A similar backbone fluctuation is also found for SBD-β and SBD-α except that they reached initial converging states at 2 and 2.5 ns respectively. In addition, the backbone RMSD of NBD got converged after 7.7 ns and remained steady for the remaining part of simulation. Besides, a high backbone RMSD (1.75 nm, at 15 ns) was found for C-Terminal part of ICM-cHSP70. This is due to the absence of C-Terminal part in the template structure of DnaK of *E*. *coli* [40]. Further, the backbone deviation for C-terminal was found higher than that for other subunits of ICM-cHSP70 in the initial 15 ns. During this phase, the unstructured C-Terminal part was trying to optimize to a folded structure and obtained it after 75ns time of simulation. Fig 4 shows that C-Terminal oscillates between the SBD-β and helix B of SBD-α and this movement after sometimes lead to unfolding event in helix C of SBD-α domain. Moreover, the behavior of “kink” found between helix A and helix B of SBD-α was more elongated in the relaxed structure than in the initial model with SBD-β being rotated (Fig 4). The behavior of atomic fluctuation was observed by calculation of RMSF of each atom of ICM-cHSP70,which provides a clear picture of atomic fluctuation for different domains and subunits of the structure, namely, domain V of NDB, Linker, Helix C of SBD-α and C-Terminal (Fig 2c). This may be due to (i) non-availability of regions in template and (ii) regions trying to optimize during simulation. In a broader view, the RMSF plot of ICM-cHSP70 show in Fig 2c indicates a similar pattern of atomic fluctuation as found in IOM-cHSP70 but with less variation in RMSF. This reveals that the close state model is more stable than open state model. Besides, the ICM-cHSP70 got converged within 30ns and optimized till end of the simulation. A 3D representation of all faces of the average stable structure is given in Fig 4.

### Analysis of compactness of cHSP70 protein

#### Open state model

The level of compactness or folding rate of IOM-cHSP70 was calculated by radius of gyration (R_g_). Considering the atoms of IOM-cHSP70in a 3D-space, the R_g_ of the systems was computed by the following formula:

$$\text{Rg}=\sqrt{\sum m_{i}||r_{i}|{|}^{2}{/}_{m_{i}}}$$ (4)

where *m*_*i*_ is the mass of i^th^ atom and *r*_*i*_ the position of atom with respect to the center of mass of the molecule [46]. Up to 20ns of time, the folding rate of the IOM-cHSP70 was found high and a maximum value of 2.70 nm was observed at 18.6ns (Fig 2d). This initial high folding rate indicates more fold recognition insight in different domains, *viz*., NBD, SBD and C-Terminal. After 20ns, the IOM-cHSP70 structure was optimized between 29ns to 30ns and the R_g_ reached minimum (structure not shown). Later on an exponential increment in the Rg was observed up to 50ns indicating major folding and unfolding events in the IOM-cHSP70. After 50 ns, a linear decrement in R_g_ (Fig 2d) was observed till the end of the simulation. Our findings were found to be in line with the earlier findings made in the protein folding [47].

#### Close state model

In case of ICM-cHSP70, initially a zigzag fashioned trend in Rg was observed but reached a maximum value of 2.0 nm at 27 ns (Fig 2d), which indicates occurrence of higher folding and unfolding events in ICM-cHSP70 subunits. Later on level of compactness of the structure was decreased in a zigzag fashion till 65ns time (Fig 2d). After this point of time Rg remained stable with minimum values indicating the higher level of compactness of the structure till end of the simulation.

### Principal component analysis of cHSP70

#### Open state model

**Fig 5:**
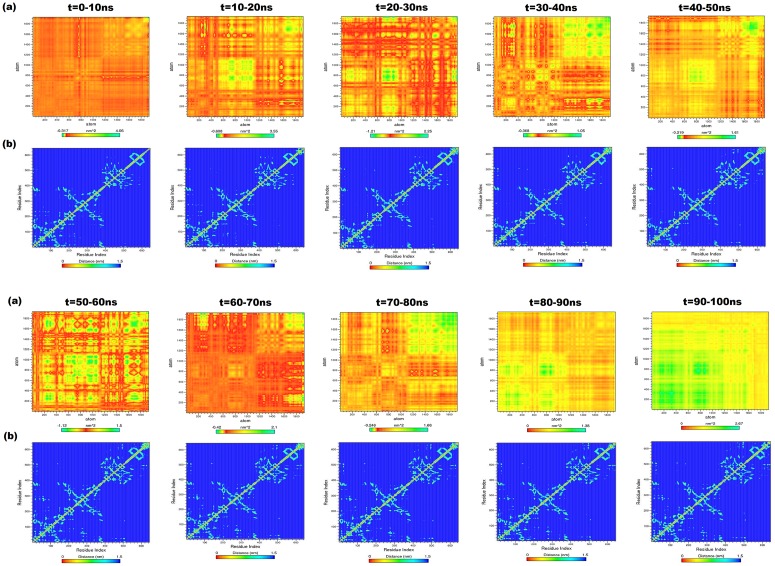
(a) Heat maps for anti-correlated and correlated motions between the backbone atoms of every successive 10ns intervals for IOM-cHSP70. The green color clusters showing the atoms moving together, the yellow color cluster showing mediatory motion and red colored clusters represent residues moving in opposite directions. The range of length for each map was depicted in indication bar. (b) The contour map for every successive interval for the trajectories of IOM-cHSP70. The diagonal line represents the zero distances between the residues paired with themselves, while cyan color spot represents the spinning motion distance (nm) for each residue pair during the course of simulation interval.

The PCA was performed on the generated trajectory of IOM-cHsp70 to monitor overall strenuous of backbone. The diagonal covariance matrix was built for 1923 backbone atoms of the protein and is used to capture the degree of co-linearity in atomic positions. Here, the covariance matrix was predicted to know whether a pair of residues has a correlated or anti-correlated motion during each successive 10ns intervals of protein trajectory. However, the eigenvalues obtained by diagonalization of covariance matrix elucidates the atomic contribution of motion. Similarly, the eigenvectors explain a collective motion accomplished by the atoms. In Fig 5a, green color clusters represent the residues moving together during the course of simulation. Whereas, the yellow color clusters indicate moderately correlated motions and the red color clusters correspond to residues that move opposite to each other. The diagonal of each interval graph (Fig 5a) represents the RMSF as measured in earlier studies [48]. The overall flexibility was calculated from the trace of diagonalized covariance matrix. The trace values that vary according to the backbone fluctuations of the IOM-cHSP70 for each time interval are given in Table 3. Among these values, higher values indicate high escalation in the flexibility with respect to native model. The highest trace value is found during 10-20ns course of simulation and can be seen in the heat map (Fig 5a), where maximum anti-correlated movement between residues is found. This indicates that structure is highly unstable and trying to change its state (open to close state). In the middle of 20-30ns time of simulation, the structure has attained the closed form and this is supported by a lower trace value. The trace value got further lowered down during 30–40 ns and reached minimum during 40–50 ns, which indicates minimum escalation in the flexibility with respect to initial model. Again a maximum trace value was observed during 50–60 ns indicating major structural change (start of lid opening). Subsequently, a minimum trace value was found during 60–90 ns, indicating a minimum escalation in the backbone flexibility. Thus a state change event from close to open state of IOM-cHSP70 is observed. Finally, during 90–100 ns a correlated movement between backbone atoms of IOM-cHSP70 was observed. The above said functional motion analysis provides a clear cut insight of the happenings in the protein during MD simulation.

**Table 3:** Ranges of heat maps and trace values for generated NxN at every successive 10ns time intervals in case of ICM-cHSP70 and IOM-cHSP70.

Interval	Range for Heat map (Min-Max) in nm^2^ for (IOM-cHSP70)	Trace value of IOM-cHsp70	Range for Heat map (Min-Max) in nm^2^ for (ICM-cHSP70)	Trace value of ICM-cHsp70
0-10ns	-0.317–4.060	1506.43	-0.758–6.640	1502.960
10-20ns	-0.608–3.550	2040.65	-0.253–1.150	1299.450
20-30ns	-1.210–2.250	1325.45	-0.791–1.730	1205.990
30-40ns	-0.368–1.050	1157.15	-0.368–1.290	684.259
40-50ns	-0.219–1.610	822.247	-0.000–1.490	1282.640
50-60ns	-1.120–1.500	4326.24	-0.0496–1.100	670.252
60-70ns	-0.420–2.100	831.732	-0.213–0.999	615.851
70-80ns	-0.246–1.680	1288.59	-0.264–2.460	1788.83
80-90ns	0.000–1.380	1053.82	-0.0879–0.509	506.118
90-100ns	0.000–2.670	2748.96	-0.266–0.723	542.564

#### Close state model

**Fig 6:**
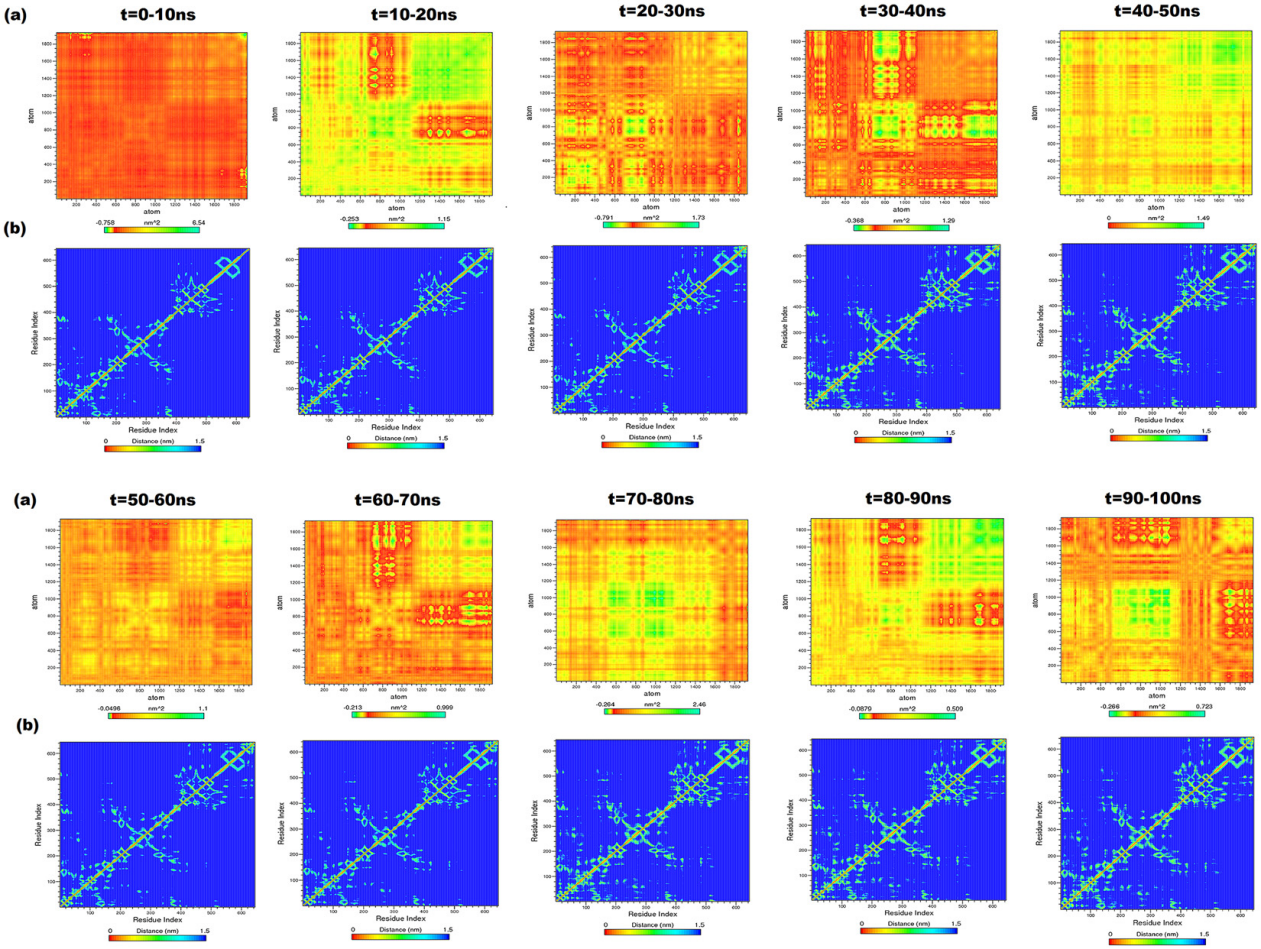
(a) Heat maps for anti-correlated and correlated motions between the backbone atoms of every successive 10ns intervals for ICM-cHSP70. The green color clusters showing the atoms moving together, the yellow color cluster showing mediatory motion and red colored clusters represent residues moving in opposite directions. The range of length for each map was depicted in indication bar. (b) The contour map for every successive interval for the trajectories of ICM-cHSP70. The diagonal line represents the zero distance between the residues paired with themselves while cyan color spot represents the spinning motions distance (nm) for each residue pair, during the course of simulation interval.

The sequence of major events of inter-domain communication for ICM-cHSP70 was analyzed by PCA for every successive 10ns interval time from the covariance matrix built for the backbone atoms of ICM-cHSP70. The diagonal of each heat map, generated for each interval trajectory, represents the RMSF for the backbone of ICM-cHSP70 (Fig 6a). A similar experiment was performed by Hunenberger et al.(1995) [48] to understand the protein motion through MD simulation. The diagonalization of covariance matrix for each interval generates a trace value (Table 2) that is directly proportional to backbone fluctuations. Here, a higher trace value was found till 30 ns, which reveals the transitional changes in the backbone as well as in different subunits of ICM-cHSP70. Subsequently, during 30-40ns the structure obtained the minimum backbone fluctuation (Fig 6a). This finding also verifies the rate of folding or level of compactness (Rg ≈ 0.4 nm). Later on during 40-50ns a sharp hike in the trace values was found, which indicates a higher rate of folding and unfolding events. This further indicates a higher inter-domain communication between SBD and NBD domains. A similar inter-domain communication behavior is found during 50–60 and 60–70 ns, except in C-Terminal (Fig 6a). The heat map of 70-80ns shows a major anti-correlated movement between the atoms of domain I and II of NBD as well as in Helix C of SBD-α and C-Terminal where as a correlated movement was found between linker and SBD-β domain. Finally, high correlated movements between atoms of different domains have been observed during 80-100ns indicating the reach out of stable structure.

### Minimum Distance Matrix calculation of cHSP70 protein

#### Open state model

The structural changes of IOM-cHSP occurred after every successive interval of 10ns simulation time and generated through *g_mdmat* program are given in Fig 5b. The diagonal line in each contour map represents zero distances between the residues with themselves, while the yellow points represent sheets which are either parallel or perpendicular to diagonal and dark spots indicate the helices. The NBD (residues 1–382) contains five sub domains (I, II, III, IV and V) (Fig 1a) and each domain has its own function during the hydrolysis of ATP to ADP [12]. Here, we have investigated the structural movements of different domains during each interval time through MDM. Initially, domains of NBD shrunk between 0-20ns and during this phase structural reformation between these domains was observed (start of lid closing event). Subsequently, the domains were fully shrunk during 20–30 ns. This inter-domain communication of NBD has directed the ‘lid’ closing event, which can be seen by viewing the first three contour maps of Fig 5b. The contour map corresponding to 20–30 ns has less variation shown by diversified the perpendicular cyan spots (for 382 NBD residues) in comparison to others. Later on the domain expansion started up to 50ns, where the map of 40-50ns time interval has similarity with the 10-20ns time interval map. After 50ns the spinning motion of residues of helices, sheets and other component of NBD got reduced till 60ns and similar spinning motion is observed in the remaining contour maps of the intervals, *viz* 60-70ns, 70-80ns and 80-90ns. The contour map of 90-100ns interval showed low spinning, *i*.*e*., minimum distance between residues. This analysis shows how different the domains of NBD (mainly I, II and IV) communicate with each other and directs the ‘lid’ opening and closing events. However, a high variation in linker (residues 383–398) is observed in the initial contour maps and become conserved at the end. The SBD-β (residues 399–509) contains eight beta sheets shown in small rectangular box and shown as cyan color spots having minimum perpendicular distance from diagonal line of the maps. The visual analysis of the contour maps at different time intervals reveals that SBD-β residues have a constant minimum distance. This shows that the SBD-β is stable during simulation. However, the SBD-α (consists of A B and C helices) shows high variability in each time interval map due to the ‘lid’ opening and closing events directed by NBD. The maximum structural changes were observed in C-Terminal (residues 616–641) as depicted in each time interval map. Finally, the MDM analysis provides overall structural movement and insight into the inter-domain communication of IOM-cHSP70. These findings are in line with those observed in HSP70 and HSP110 of other species [49, 50].

#### Close state model

The Fig 6b shows the contour maps of residue movement of ICM-cHSP70 trajectory for every successive 10ns interval. The spinning motion of residues for NBD and its sub-domains followed a similar pattern till 30ns interval time. However, the residues are in a spinning motion during 30-50ns interval time indicating a higher inter domain communication or domain rotation that leads to ‘lid’ opening event. Later on, the NBD domain again relaxed and showed a similar pattern in distance matrix during of 50-60ns and 60-70ns time intervals (Fig 6b). In addition, the start of anti-correlated motions were found between the residues of NBD during 70-100ns that directs the expansion of NBD sub-domains and lead to ‘lid’ closing event. However, the behavior of linker (383–398) does not change much (Fig 6b). The SBD-β domain of ICM-cHSP70 followed a similar pattern as seen in the case of IOM-cHSP70, *i*.*e*., close to open state and vice-versa transitions. Whereas the residue movement in helices of SBD-αfound to be correlated during ‘lid’ opening (the kink of B and C helices became straight)but found to be anti-correlated during ‘lid’ closing event. These results corroborates with the findings of Chiappori, et al. (2012) and Liu & Hendrickson (2007) [49, 50]. Besides, a maximum structural change was observed in the C-Terminal (residues 616–641) of ICM-cHSP70, similar to that found inIOM-cHSP70 model.

### Conformational changes between open and close states of cHSP70

**Fig 7:**
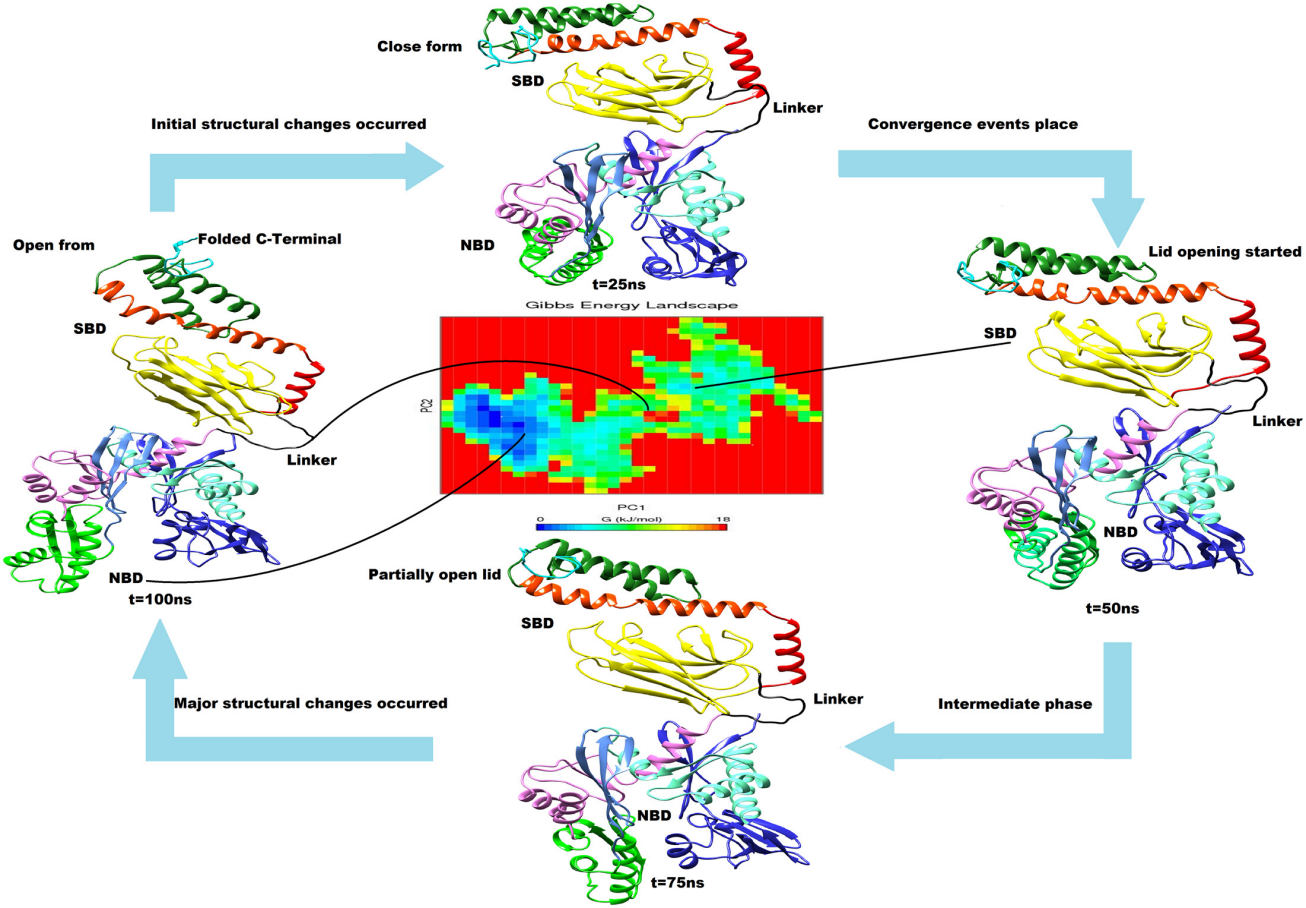
Structural and functional identification of cHSP70 structure during simulation having similar color code as defined in Fig 1a. The averaged structure for each interval with structural changes was shown. Gibbs free energy landscape represents the frequency of subunit folding identified via Principal Component Analysis (PCA). The minimum Gibbs free energy (blue color spot) shows minimum folding rates in domains while the intermediate and higher energy (green and yellow spots) shows intermediate and higher folding rates in subunits of cHSP70.

The relaxed models of cHSP70 describe the initial and final structures as well as conformational evolution of the chaperon functioning cycle of the protein. The close state to open state changes are, in general, directed by the nucleotide exchange rate and being initiated by hydrolysis of ATP to ADP in reaction solution and *vice versa* [51]. The nucleotide exchange rate and hydrolysis of ATP were increased by several folds through interaction of other co-chaperones and binding of the substrate or polypeptide to SBD [40, 52]. We have identified functionally important inter domain communication motion for transition between open state to close state and close state to open state of cHSP70 by applying the PCA technique. A Similar collective mode motion analysis for studying conformational changes in protein using standard normal mode (NMA) technique was given by Tama, et al. (2001) [29] with certain limitations. Here, we have opted PCA technique to overcome the limitations associated with the standard NMA. Hence, the collective modes of the motion and their relevance to open state to close state and *vice versa* for cHSP70 were calculated. The transitions between different states for cHSP70 identified during simulations are given in Fig 7 as well as presented through a video (S1 Video). The figure and video shows how functionally active NBD, SBD and C-Terminal are participating in conversion of open state to close state and *vice versa*. From the video one can observe the important functional movement of different domains of NDB which simultaneously performed the ‘lid’ opening and closing events of SBD and role of C-Terminal. Although we could not perform the hydrolysis of ATP to ADP and ADP to ATP reactions computationally due to limitation of resources, the shrinking of NBD initiated by the hydrolysis of ATP that directs the ‘lid’ opening (close to open state of cHSP70) and expansion of NDB, initiated by the presence of ADP and inorganic phosphorous (P) that directs the ‘lid’ closing (open to close state of cHSP70) can be observed in S1 Video.

### Peptide modeling and docking

**Fig 8:**
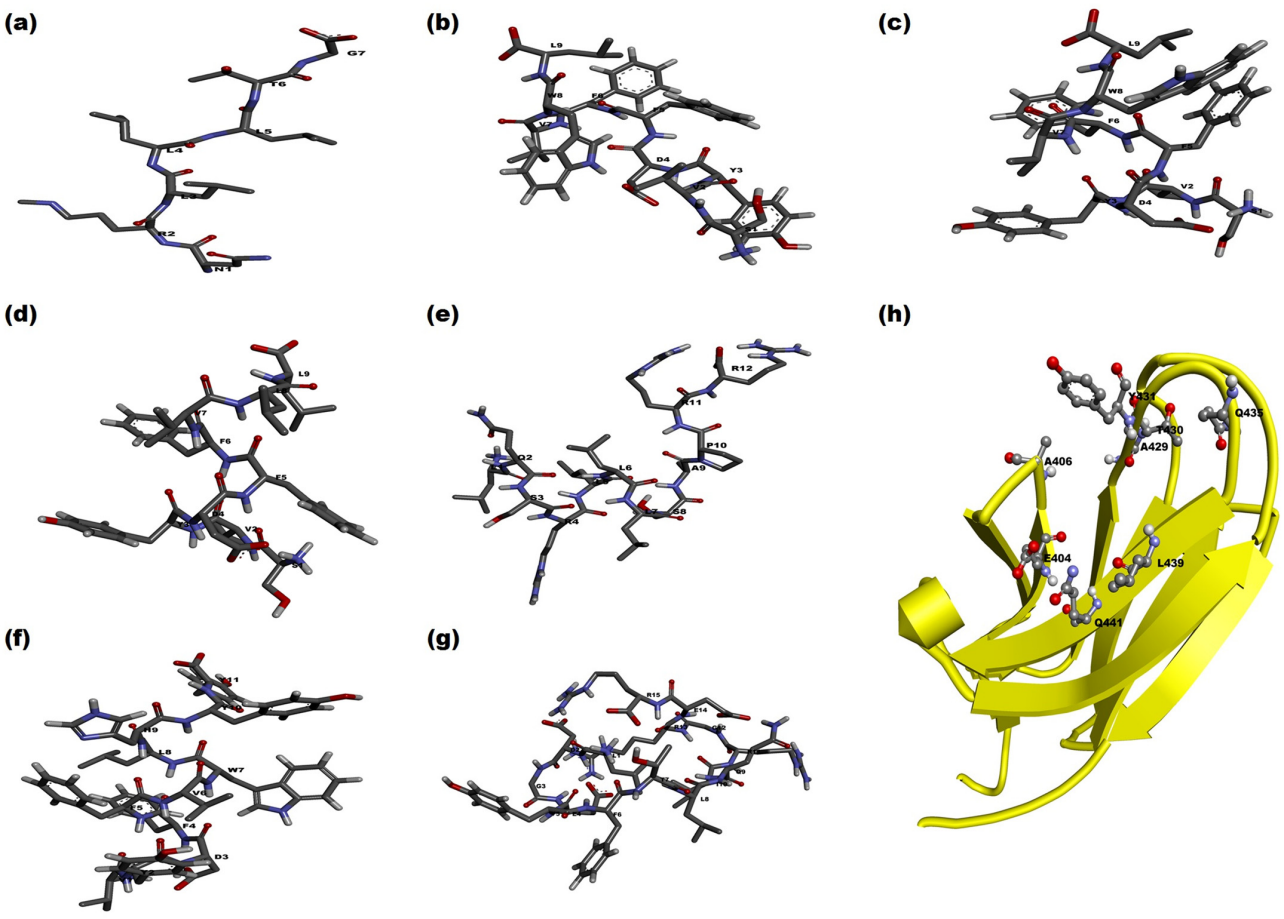
Stick representation of 3D modeled structure of different peptide substrates namely (a) NR, (b) TRP2, (c) TRP_F5L/F6L, (d) TRP2_W8L, (e) p12, (f) TRP2_181, (g) p53.(h) Cartoon representation of SBD-β with substrate binding residues shown as ball and stick fashion.

**Fig 9:**
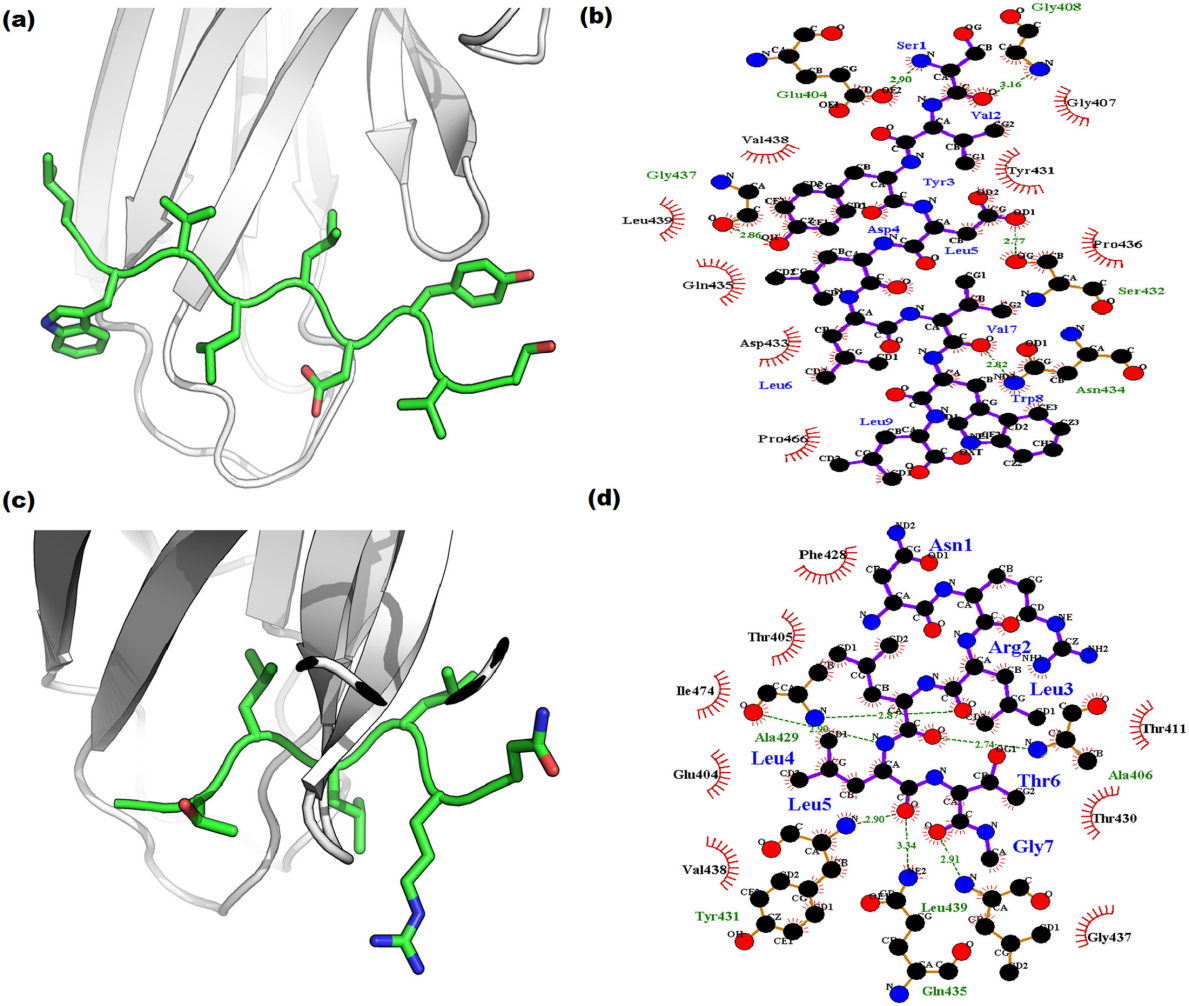
Cartoon repersentation of dockedcomplexes. (a) SBD-β with TRP2_F5L/F6L (c) SBD-β with NR. The molecular interaction plots: (b) SBD-β with TRP2_F5L/F6L (d) SBD-β with NR. The complexesare generated via LIGPLOT.

The 3D structures of seven peptides (Table 1) modeled through PEP-FOLD and PEPSTR servers with the SBD-β domain are given in Fig 8. Both servers have generated the peptide models and performed energy minimization. We have selected the most stable peptide model and docked into the simulated SBD-β domain to establish the interaction between them. The stability of interactions depends on nature of catalytic site as well as peptides that are hydrophobic or hydrophilic and positively or negatively charged in nature. The SBD-β contains a hydrophobic groove between outer and inner loops which interact with substrate or peptide to perform the chaperon activity. The Z-DOCK results have provided a number of docked protein-peptide complexes. Evaluation of docking complexes was done on the basis of protein-peptide compatibility in terms of Hydrogen bonding, Van dar Waal interaction, Electrostatic interaction and satiric interaction. According to the stability of complex, the Z-DOCK has calculated the Z-score. Form Z-Score, the highest ranked complex was selected for protein-peptide analysis. The interaction analysis revealed that most of the peptides have formed a similar conformation in the cavity of SBD-β (Table 4). The details of interacting residues for each peptide complex are given in Table 4. The TRP2 and TRP2_W8L peptides have shown better compatibility (*in-silico*) with protein and the same is evident from LIGPLOT interaction plots of TRP2 and TRP2_W8L (S3 Fig). The best complex of each peptide was further refined in Rosetta FlexPepDock server that provides high resolution and full flexibility to peptide backbone and the protein receptor site to interact. Best decoy for each complex out of 1000 generated decoys was identified and analyzed. The top models of refined complexes have shown better interaction stability in comparison to Z-DOCK based complexes. The peptides NR, TRP2_F5L/F6L and TRP2_181 (Fig 9) have shown better compatibility in the catalytic groove of SBD-β of cHSP70. A similar result was proved in case of HSP110 through a wet lab study carried out by Xu et al. (2012) [30]. Moreover, a comparative interaction analysis performed between binding sites of SBD- β participated during docking and complex refinements are given in Table 4. This analysis reveals that residues Ala 406, Ile427, Ala429, Gly437 and Leu439 of SBD-β of cHSP70 have participated in the hydrogen bonds formation during all peptides docking and their refinement process. The actively participated common residues in the formation of Van der Wall and Electrostatic interactions are Glu404, Thr405, Ala406, Val409, Gln426, Phe428, Thr430, Tyr431, Ser432, Gly437, Val438, Leu439, Glu460, Leu461, Ile464 and Pro466. This docking interaction analysis also confirmed the substrate binding groove of cHSP70. Such substrate binding groove in HSP70 and other proteins were reported earlier in other species [53, 54]. Moreover, to confirm the presence of the above said active sites in other animal HSP70 protein expressed by HSPA1A gene, we performed MSA of 17 selected sequences (S4 Fig). Result shows that all the catalytic grove residues are similar in animal species like Cow, Sheep, Buffalo and Goat (expect Ala429 of Indian camel).

**Table 4:** Comparative analysis of binding site residues of SBD-β of cHSP70 participating in docking and their refinement from Z-Dock and FlexPepDock servers.

Name of Protein-peptide complex	Z-Dock Output		FelxPepDock refinement Output	
	Hydrogen bonds forming residues of SBD-β	Cavity residues of SBD-β (forms other types bonds)	Hydrogen bonds forming residues of SBD-β	Cavity residues of SBD-β (forms other types bonds)
SBD-β and NR	Ala 406, Ile427, Ala429, Gly437	Glu404, Thr405, Ala406, Phe428, Thr430, Gly437, Val438, Leu439, Arg469, Val476	Ala 406, Ala429, Tyr 431, Gln435, Leu 439	Glu404, Thr 405, Thr411, Phe 428, Thr430, Gly437, Val438, Ile474
SBD-β and TRP2	Ser 432, Gln435, Pro436, Gly437 Leu 439,Gln441, Ser462	Glu404, Thr405, Ala406, Gly407, Tyr431, Asp433, Gln435, Val438, Leu439 Leu461, Gly463, Ile464, Pro466	Ser 432, Gln435, Gly437 Leu 439,	Glu404, Thr405, Ala406, Gly407, Tyr431, Asp433, Gln435, Val438, Leu439 Arg458, Glu460, Leu461, Gly463, Ile464, Pro466
SBD-β and p53	Ile427, Ala429	Glu404, Thr405, Ala406, Gly407, Gln424,Thr425, Phe428, Thr430, Tyr431, Asp433, Gln435, Val438, Leu439 Leu461, Gly463, Ile464, Pro466	Ala 406, Ala429, Tyr 431, Gln435, Glu460	Leu403, Glu404, Thr 405, Thr411, Phe 428, Thr430, Gly437, Val438, Leu439,Ile440 Ile474
SBD-β and p12	Ile427, Ala429	Glu404,Thr405, Ala406, Gln424, Thr425, Gln426, Phe428, Thr430, Tyr431, Ser432, Val438, Leu461 Gly463, Pro466, Gly470	Ala 406, Ala429, Tyr 431, Leu439,	Glu404,Thr405, Ala406, Val409, Thr411, Gln426, Phe428, Thr430, Tyr431, Ser432, Gly437,Val438,
SBD-β and TRP2_F5L/F6L	Tyr 431,Ser432 Gly437 Leu439, Gln441, Ser462	Glu404,Thr405, Ala406, Gly407 Thr425, Gln426, Phe428, Thr430, Tyr431, Asp433, Gln435, Val438, Glu460, Leu461 Gly463, Ile464, Pro466, Gly470	Glu404, Gly408,Ser432, Asn434, Gly437,Leu439	Gly407,Gln426, Phe428, Thr430, Tyr431,Asp433, Gln435, Val438, Leu439, Glu460, Leu461 Gly463, Ile464, Pro466
SBD-β and TRP2_W8L	Ala412, Gln426, Ile427 Ala429, Tyr 431, Ser432, Gly437, Leu439, Arg458	Leu 403,Glu404, Thr405, Thr411, Leu413, Tyr431, Asp433,Gly437, Gln435, Val438, Ile440, Glu460, Leu461 Gly463, Ile464, Pro466, Gly470, Gln473, Ile474	Ala.406, Gln426, Ala429,Arg 469	Gly407,Gln426, Phe428, Thr430, Tyr431,Asp433, Gln435, Val438, Leu439, Glu460, Leu461 Gly463, Ile464, Pro466
SBD-β and TRP2_181	Ala406, Ala429 Gly437, Leu439	Glu404,Thr405, Val409 Gln426, Ile427 Phe428, Ala229, Thr430, Tyr431 Asp433,Gly437, Gln435, Val438, Ile440, Gln441 Glu460, Leu461, Ser460, Gly463, Ile464, Pro466, Gly470	Ala406, Ala429 Tyr431, Gln435, Gly437, Leu439	Glu404, Thr405, Gln426, Ile427 Phe428, Ala229, Thr430, Tyr431 Asp433,Gly437, Gln435, Val438, Ile440, Gln441 Glu460, Leu461 Gly463, Ile464, Pro466, Gly470

## Conclusions

The prime objective of this work was to construct stable 3D models of cHSP70 under open and closed states in absence of other co-chaperones. Two steps of the chaperon cycle of cHSP70 correspond to two typical structures of cHSP70 under open and close states. The structures were generated through multi-template comparative modeling and further relaxed using all-atom MD simulations at constant temperature (300 K) and pressure (1bar). We have obtained the stable structures for IOM-cHSP70 and ICM-cHSP70 models in similar environmental conditions. The open state model adopts a stable conformation in which the ‘lid’ remains in open state whereas in closed state the ‘lid’ is closed. Moreover, an insight into the inter domain communication during the state transition was explained through PCA and MDM analyses. Both the analyses were performed for every 10ns time intervals during MD simulation to precisely identify the conformational and functional changes in the structure. The IOM-cHSP70 provides a similar dynamic behavior reported earlier by different groups. However, the different transition states of inter domain communication via collective mode analysis has shown that the opening of substrate binding pocket causes a simultaneous expansion of NBD subdomains. Whereas, shrinking in NBD subdomains cause closure of substrate binding pocket. Further, the catalytic groove of SBD-β in cHSP70 was identified through protein-peptide docking analysis. The present study carried out in cHSP70 may be helpful to analyze the chaperon cycle of HSP70 in other animals.

## Supporting Information

S1 Fig Representation of secondary structure elements and predicted domains for (a) IOM-cHsp70 and (b) ICM-cHsp70 by Pro-Func server. (TIF)

S2 Fig Neighbor joining phylogenetic tree with bootstrap values forHSP70 1A protein expressed under HSPA1A genes. Total 33 branches are classified into three main groups: first group marked with blue line having more similarities with camel HSP70 1A, second group marked with red line and contain 5 branches of Mouse, Rat and Hamster and third group marked as green line with more diverse branches as compared to other two groups.
(TIF)

S3 Fig Ligplot molecular intraction plots for (a) SBD-β and TP2_W8L (b) SBD-β and TRP2generated after ZDOCK docking. (TIF)

S4 Fig Multiple sequance alignment (MSA) for 17 selcted animal HSP70 protein sequance expressed by HSPA1A/HSPA1B gene. The black rectangular boxes repersent the common residues forming hydrogen bonds and yellow rectangualar boxes represent the residues present in active sites of SBD-β.
(TIF)

S1 Table List of sequances and their accesion number used for the comparive evolutionary study of HSP70 1A/1B protein. (DOCX)

S1 Video showing how different domains of cHSP70 comunicating with each other during all atom MD simulation. (MPG)
