# Correction: Extrapolation of Inter Domain Communications and Substrate Binding Cavity of Camel HSP70 1A: A Molecular Modeling and Dynamics Simulation Study

**DOI:** 10.1371/journal.pone.0138961

**Published:** 2015-09-18

**Authors:** The PLOS ONE Staff

Fig 4 incorrectly appears as a duplicate of Fig 5. The publisher apologizes for the error. Please find the corrected version of Fig 4 here.

**Fig 4:**
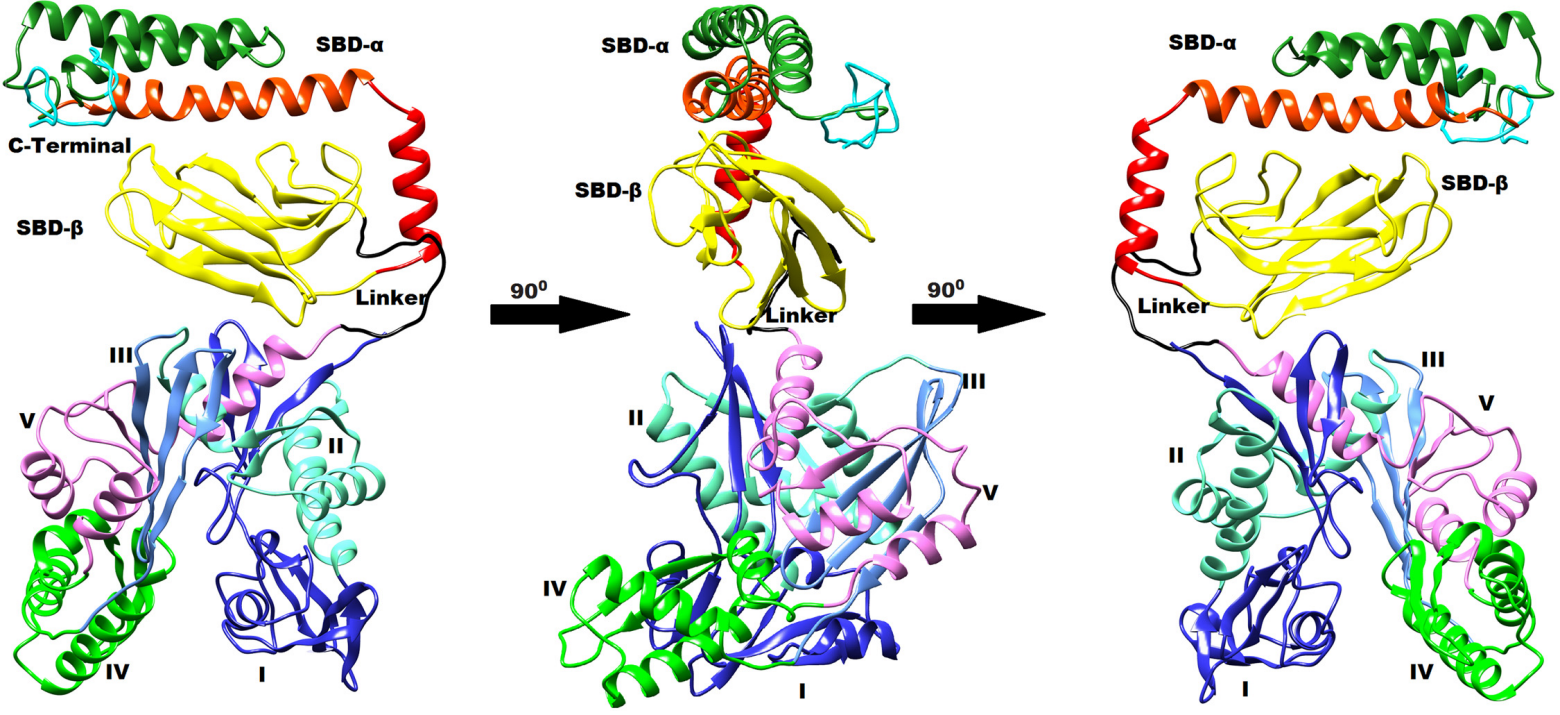
Typical average 3D structure represented in cartoon diagram of close state of cHSP70 rotated by 90° after relaxation through MD simulation.
