# Supplementary figures and images for: Extrapolation of Inter Domain Communications and Substrate Binding Cavity of Camel HSP70 1A: A Molecular Modeling and Dynamics Simulation Study
Source: PLoS One. 2015 Aug 27;10(8):e0136630. doi: 10.1371/journal.pone.0136630 (PMC4552423; doi:10.1371/journal.pone.0136630)

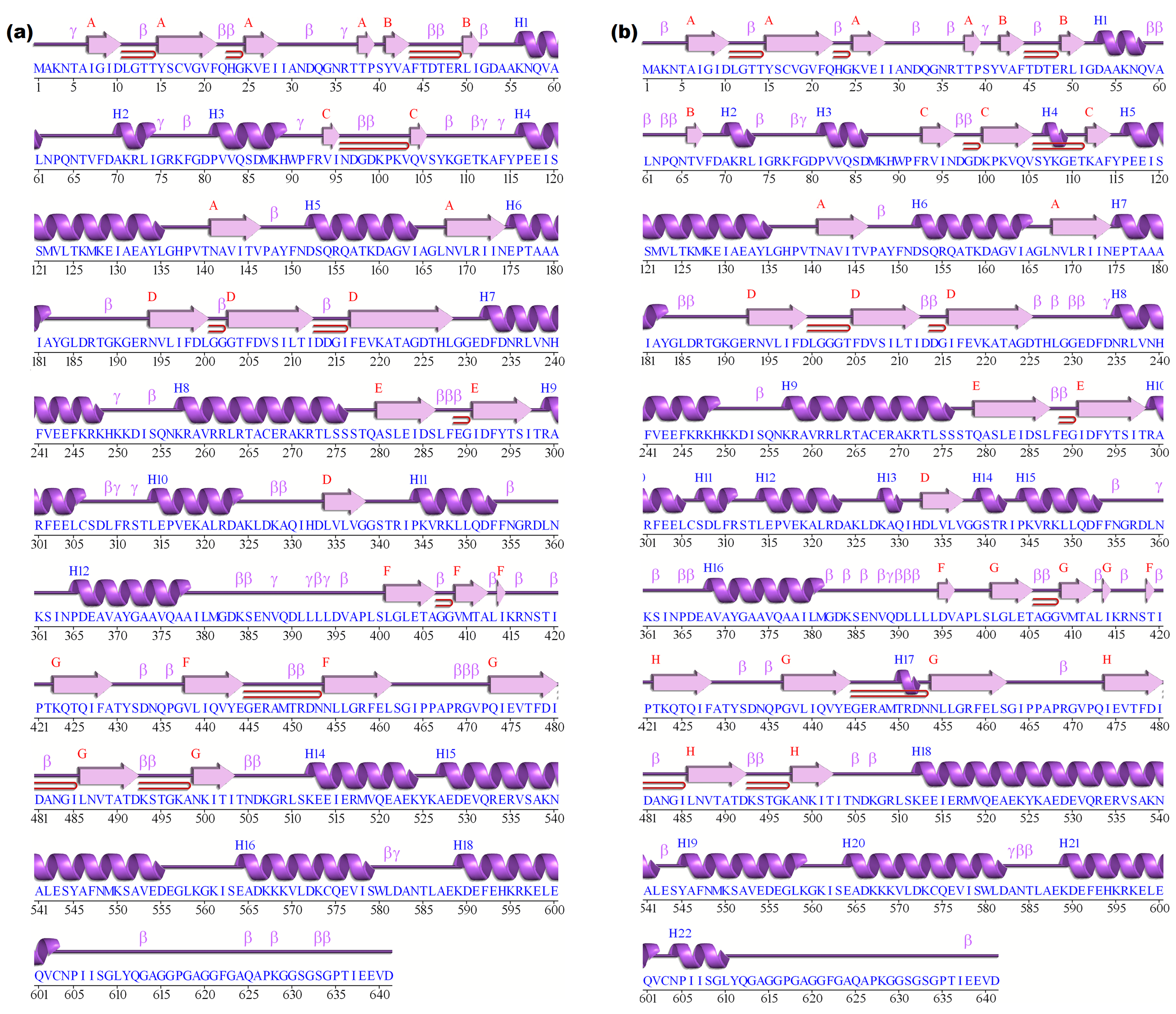

Supplement: S1 Fig — (TIF) [file pone.0136630.s001.tif]

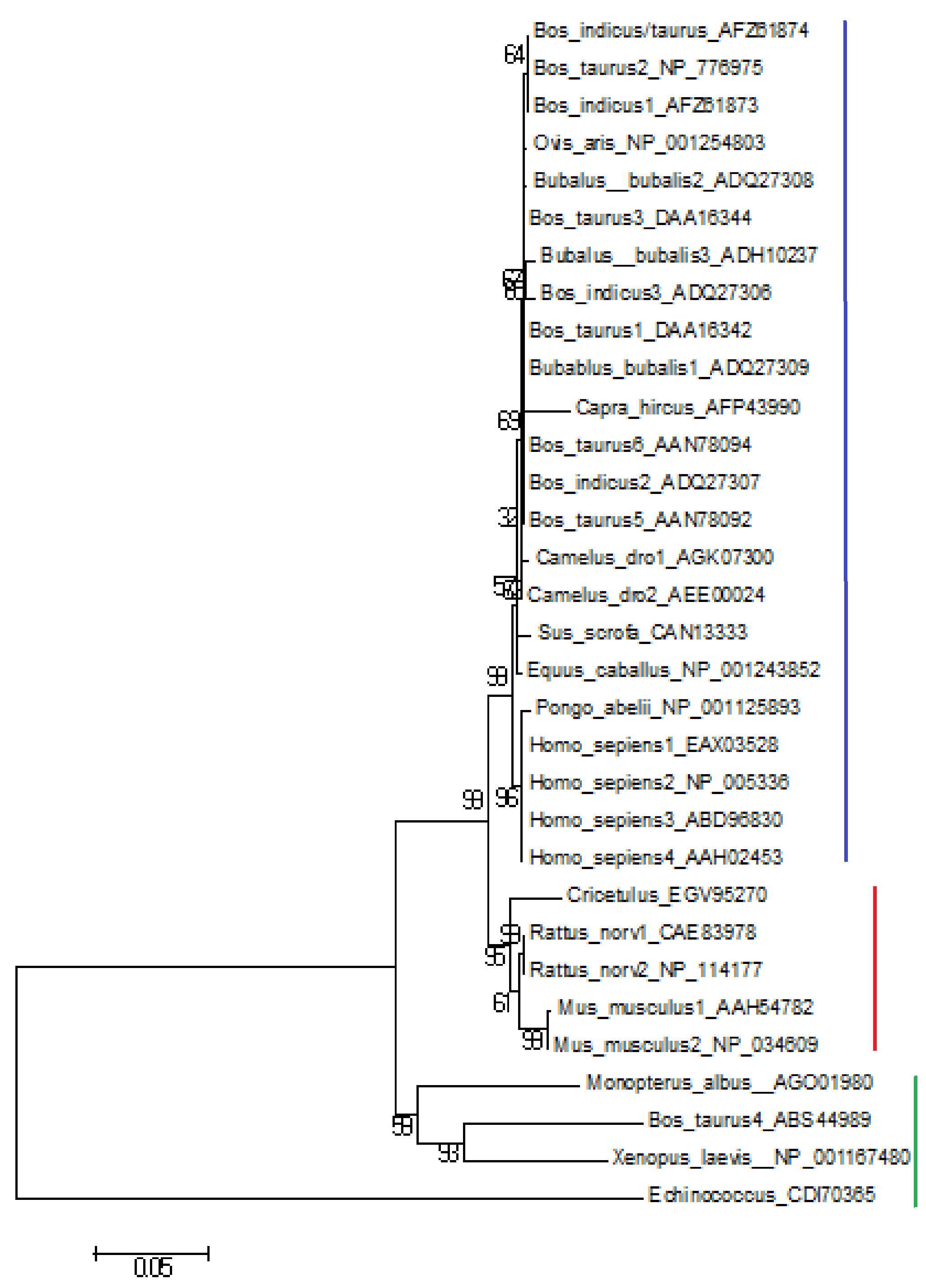

Supplement: S2 Fig — Total 33 branches are classified into three main groups: first group marked with blue line having more similarities with camel HSP70 1A, second group marked with red line and contain 5 branches of Mouse, Rat and Hamster and third group marked as green line with more diverse branches as compared to other two groups. (TIF) [file pone.0136630.s002.tif]

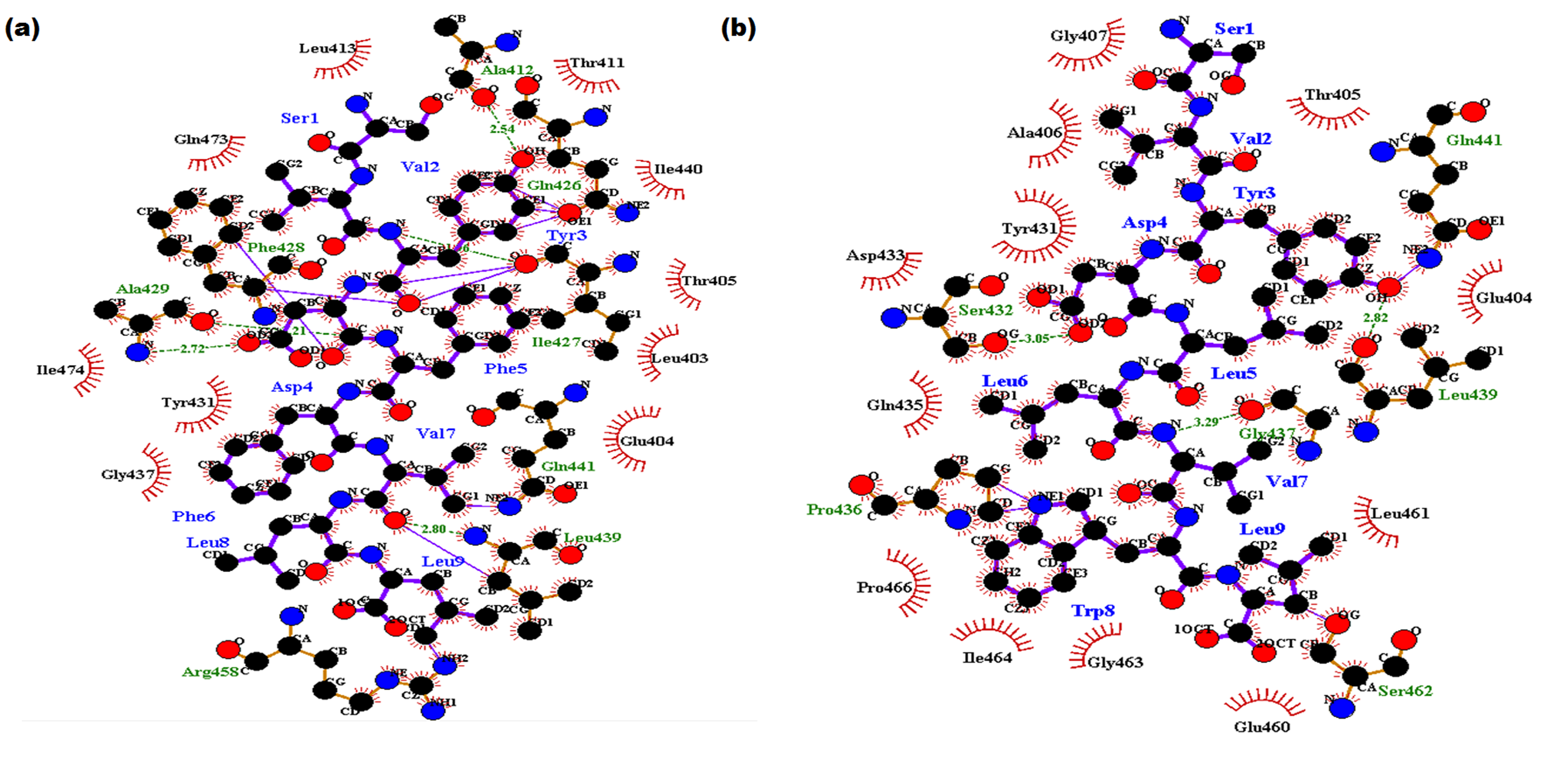

Supplement: S3 Fig — (TIF) [file pone.0136630.s003.tif]

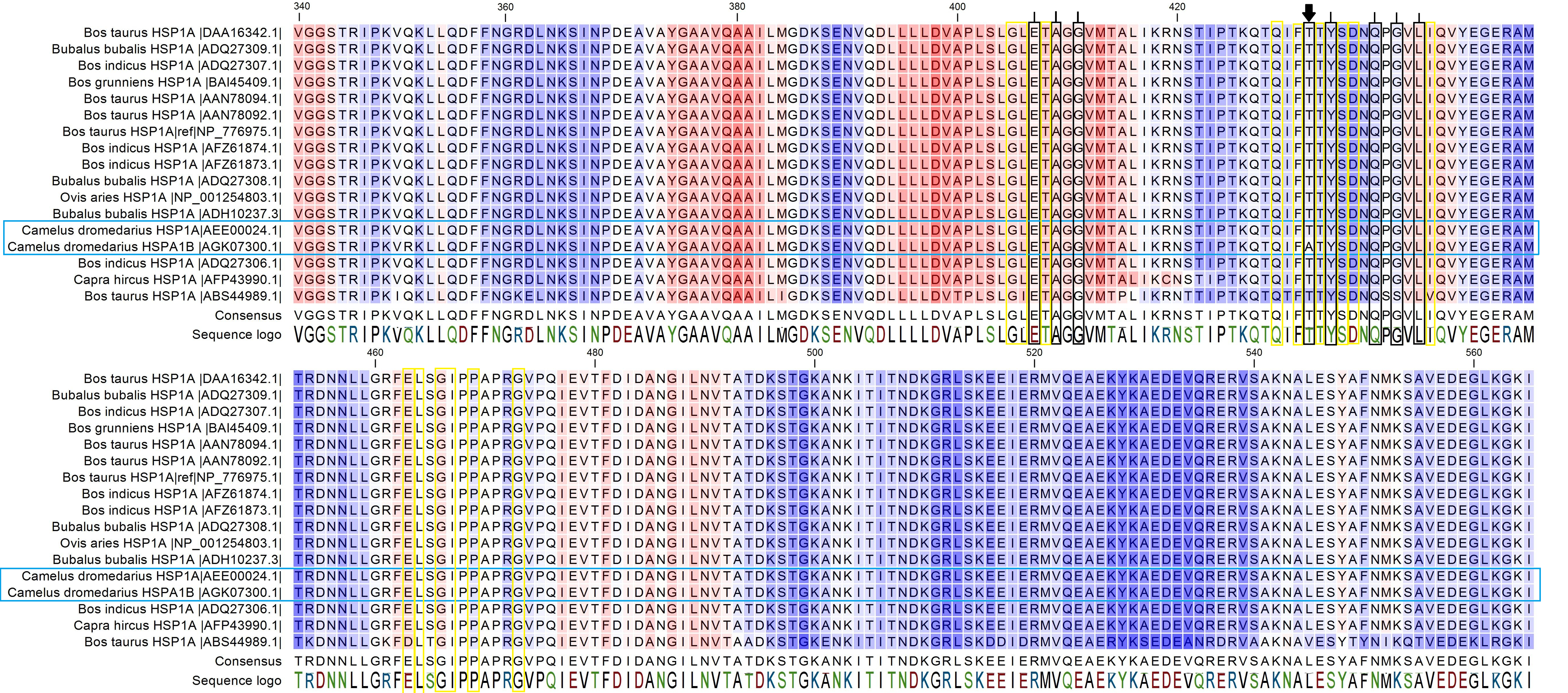

Supplement: S4 Fig — The black rectangular boxes repersent the common residues forming hydrogen bonds and yellow rectangualar boxes represent the residues present in active sites of SBD-β. (TIF) [file pone.0136630.s004.tif]
