# Supplementary material for: Extrapolation of Inter Domain Communications and Substrate Binding Cavity of Camel HSP70 1A: A Molecular Modeling and Dynamics Simulation Study
Source: PLoS One. 2015 Aug 27;10(8):e0136630. doi: 10.1371/journal.pone.0136630 (PMC4552423; doi:10.1371/journal.pone.0136630)
Supplement: S1 Table — (DOCX) [file pone.0136630.s005.docx]

S1 Table. List of sequances and their accesion number used for the comparive evolutionary study of HSP70 1A/1B protein.

| **Sl.No.** | **Accession No.** | **Sequences of HSP70 1A/1B protein used for Phylogentic tree construction** |
| --- | --- | --- |
| 1. | DAA16342.1 | >gi\|296474227\|tpg\|DAA16342.1\| TPA: heat shock 70 kDa protein 1A [*Bos taurus*]  MAKNMAIGIDLGTTYSCVGVFQHGKVEIIANDQGNRTTPSYVAFTDTERLIGDAAKNQVALNPQNTVFDAKRLIGRKFGDPVVQSDMKHWPFRVINDGDKPKVQVSYKGETKAFYPEEISSMVLTKMKEIAEAYLGHPVTNAVITVPAYFNDSQRQATKDAGVIAGLNVLRIINEPTAAAIAYGLDRTGKGERNVLIFDLGGGTFDVSILTIDDGIFEVKATAGDTHLGGEDFDNRLVNHFVEEFKRKHKKDISQNKRAVRRLRTACERAKRTLSSSTQASLEIDSLFEGIDFYTSITRARFEELCSDLFRSTLEPVEKALRDAKLDKAQIHDLVLVGGSTRIPKVQKLLQDFFNGRDLNKSINPDEAVAYGAAVQAAILMGDKSENVQDLLLLDVAPLSLGLETAGGVMTALIKRNSTIPTKQTQIFTTYSDNQPGVLIQVYEGERAMTRDNNLLGRFELSGIPPAPRGVPQIEVTFDIDANGILNVTATDKSTGKANKITITNDKGRLSKEEIERMVQEAEKYKAEDEVQRERVSAKNALESYAFNMKSAVEDEGLKGKISEADKKKVLDKCQEVISWLDANTLAEKDEFEHKRKELEQVCNPIISRLYQGAGGPGAGGFGAQGPKGGSGSGPTIEEVD |
| 2. | AFZ61873.1 | >gi\|428697029\|gb\|AFZ61873.1\| heat shock 70kDa protein 1A [*Bos indicus*]  MAKNMAIGIDLGTTYSCVGVFQHGKVEIIANDQGNRTTPSYVAFTDTERLIGDAAKNQVALNPQNTVFDAKRLIGRKFGDPVVQSDMKEWPFRVINDGDKPKVQVSYKGETKAFYPEEISSMVLTKMKEIAEAYLGHPVTNAVITVPAYFNDSQRQATKDAGVIAGLNVLRIINEPTAAAIAYGLDRTGKGERNVLIFDLGGGTFDVSILTIDDGIFEVKATAGDTHLGGEDFDNRLVNHFVEEFKRKHKKDISQNKRAVRRLRTACERAKRTLSSSTQASLEIDSLFEGIDFYTSITRARFEELCSDLFRSTLEPVEKALRDAKLDKAQIHDLVLVGGSTRIPKVQKLLQDFFNGRDLNKSINPDEAVAYGAAVQAAILMGDKSENVQDLLLLDVAPLSLGLETAGGVMTALIKRNSTIPTKQTQIFTTYSDNQPGVLIQVYEGERAMTRDNNLLGRFELSGIPPAPRGVPQIEVTFDIDANGILNVTATDKSTGKANKITITNDKGRLSKEEIERMVQEAEKYKAEDEVQRERVSAKNALESYAFNMKSAVEDEGLKGKISEADKKKVLDKCQEVISWLDANTLAEKDEFEHKRKELEQVCNPIISRLYQGAGGPGAGGFGAQGPKGGSGSGPTIEEVD |
| 3. | AFZ61874.1 | >gi\|428697031\|gb\|AFZ61874.1\| heat shock 70kDa protein 1A [*Bos indicus* x *Bos taurus*]  MAKNMAIGIDLGTTYSCVGVFQHGKVEIIANDQGNRTTPSYVAFTDTERLIGDAAKNQVALNPQNTVFDAKRLIGRKFGDPVVQSDMKEWPFRVINDGDKPKVQVSYKGETKAFYPEEISSMVLTKMKEIAEAYLGHPVTNAVITVPAYFNDSQRQATKDAGVIAGLNVLRIINEPTAAAIAYGLDRTGKGERNVLIFDLGGGTFDVSILTIDDGIFEVKATAGDTHLGGEDFDNRLVNHFVEEFKRKHKKDISQNKRAVRRLRTACERAKRTLSSSTQASLEIDSLFEGIDFYTSITRARFEELCSDLFRSTLEPVEKALRDAKLDKAQIHDLVLVGGSTRIPKVQKLLQDFFNGRDLNKSINPDEAVAYGAAVQAAILMGDKSENVQDLLLLDVAPLSLGLETAGGVMTALIKRNSTIPTKQTQIFTTYSDNQPGVLIQVYEGERAMTRDNNLLGRFELSGIPPAPRGVPQIEVTFDIDANGILNVTATDKSTGKANKITITNDKGRLSKEEIERMVQEAEKYKAEDEVQRERVSAKNALESYAFNMKSAVEDEGLKGKISEADKKKVLDKCQEVISWLDANTLAEKDEFEHKRKELEQVCNPIISRLYQGAGGPGAGGFGAQGPKGGSGSGPTIEEVD |
| 4. | NP_776975.1 | >gi\|40254806\|ref\|NP_776975.1\| heat shock 70 kDa protein 1A [*Bos taurus*]  MAKNMAIGIDLGTTYSCVGVFQHGKVEIIANDQGNRTTPSYVAFTDTERLIGDAAKNQVALNPQNTVFDAKRLIGRKFGDPVVQSDMKEWPFRVINDGDKPKVQVSYKGETKAFYPEEISSMVLTKMKEIAEAYLGHPVTNAVITVPAYFNDSQRQATKDAGVIAGLNVLRIINEPTAAAIAYGLDRTGKGERNVLIFDLGGGTFDVSILTIDDGIFEVKATAGDTHLGGEDFDNRLVNHFVEEFKRKHKKDISQNKRAVRRLRTACERAKRTLSSSTQASLEIDSLFEGIDFYTSITRARFEELCSDLFRSTLEPVEKALRDAKLDKAQIHDLVLVGGSTRIPKVQKLLQDFFNGRDLNKSINPDEAVAYGAAVQAAILMGDKSENVQDLLLLDVAPLSLGLETAGGVMTALIKRNSTIPTKQTQIFTTYSDNQPGVLIQVYEGERAMTRDNNLLGRFELSGIPPAPRGVPQIEVTFDIDANGILNVTATDKSTGKANKITITNDKGRLSKEEIERMVQEAEKYKAEDEVQRERVSAKNALESYAFNMKSAVEDEGLKGKISEADKKKVLDKCQEVISWLDANTLAEKDEFEHKRKELEQVCNPIISRLYQGAGGPGAGGFGAQGPKGGSGSGPTIEEVD |
| 5. | BAI45409.1 | >gi\|261857774\|dbj\|BAI45409.1\| heat shock 70kDa protein 1A, partial [synthetic construct]  MAKAAAIGIDLGTTYSCVGVFQHGKVEIIANDQGNRTTPSYVAFTDTERLIGDAAKNQVALNPQNTVFDAKRLIGRKFGDPVVQSDMKHWPFQVINDGDKPKVQVSYKGDTKAFYPEEISSMVLTKMKEIAEAYLGYPVTNAVITVPAYFNDSQRQATKDAGVIAGLNVLRIINEPTAAAIAYGLDRTGKGERNVLIFDLGGGTFDVSILTIDDGIFEVKATAGDTHLGGEDFDNRLVNHFVEEFKRKHKKDISQNKRAVRRLRTACERAKRTLSSSTQASLEIDSLFEGIDFYTSITRARFEELCSDLFRSTLEPVEKALRDAKLDKAQIHDLVLVGGSTRIPKVQKLLQDFFNGRDLNKSINPDEAVAYGAAVQAAILMGDKSENVQDLLLLDVAPLSLGLETAGGVMTALIKRNSTIPTKQTQIFTTYSDNQPGVLIQVYEGERAMTKDNNLLGRFELSGIPPAPRGVPQIEVTFDIDANGILNVTATDKSTGKANKITITNDKGRLSKEEIESMVQEAEKYKAEDEVQRERVSAKNALESYAFNMKSAVEDEGLKGKISEADKKKVLDKCQEVISWLDANTLAEKDEFEHKRKELEQVCNPIISGLYQGAGGPGPGGFGAQGPKGGSGSGPTIEEVD |
| 6. | DAA16344.1 | >gi\|296474229\|tpg\|DAA16344.1\| TPA: heat shock 70 kDa protein 1A/1B [*Bos taurus*]  MAKNMAIGIDLGTTYSCVGVFQHGKVEIIANDQGNRTTPSYVAFTDTERLIGDAAKNQVALNPQNTVFDAKRLIGRKFGDPVVQSDMKHWPFRVINDGDKPKVQVSYKGETKAFYPEEISSMVLTKMKEIAEAYLGHPVTNAVITVPAYFNDSQRQATKAGVIAGLNVLRIINEPTAAAIAYGLDRTGKGERNVLIFDLGGGTFDVSILTIDDGIFEVKATAGDTHLGGEDFDNRLVNHFVEEFKRKHKKDISQNKRAVRRLRTACERAKRTLSSSTQASLEIDSLFEGIDFYTSITRARFEELCSDLFRSTLEPVEKALRDAKLDKAQIHDLVLVGGSTRIPKVQKLLQDFFNGRDLNKSINPDEAVAYGAAVQAAILMGDKSENVQDLLLLDVAPLSLGLETAGGVMTALIKRNSTIPTKQTQIFTTYSDNQPGVLIQVYEGERAMTRDNNLLGRFELSGIPPAPRGVPQIEVTFDIDANGILNVTATDKSTGKANKITITNDKGRLSKEEIERMVQEAEKYKAEDEVQRERVSAKNALESYAFNMKSAVEDEGLKGKISEADKKKVLDKCQEVISWLDANTLAEKDEFEHKRKELEQVCNPIISRLYQGAGGPGAGGFGAQGPKGGSGSGPTIEEVD |
| 7. | AFP43990.1 | >gi\|399518486\|gb\|AFP43990.1\| heat shock 70kDa protein 1A [*Capra hircus*]  MAKNMAIGIDWGTTYSCVGVFQHGKVEIIANDQGNRTTPSYVAFTDTERLIGDAAKNQVALNPQNTVFDAKRLICRKFGDPVVQSDMKHWPFRVINDGDKPKVQVSYKGETKAFYPEEISSMVLTKMKEIAEAYLGHPVTNAVITVPAYFNDTQRQATKDAGVIAGLNVLRIINEPTAAAIAYGLDRTGKGECNVLIFDLGGGTFDVSILTIDDGIFEVKATAGDTHLGGEDFDNRLVNYFVEEFTRKHKKDISQNKRAVRRLRTACERAKRTLSSSTQASLEIDSLFEGIDFNTSITRARFEEQCSDLFRSTLEPVEKALSDAKLDKAQIHDLVLVGGSTRIPKVQKLLQDFFNGRDLNKSINPDEAVAYGAAVQAAILMGDKSENVQDLLLLDVAPLSLGLETAGGVMTALIKCNSTIPTKQTQIFTTYSDNQPGVLIQVYEGERAMTRDNNLLGRFELSGIPPAPRGVPQIEVTFDIDANGILNVTATDKSTGKANKITITNDKGRLSKEEIERMVQEAEKYKAEDEVQRERVSAKNALESYAFNMKSAVEDEGLKGKISEADKKVVLDKCQEVISWLDANTLAEKDEFEHKRKELEQMCNPIISRLYQGAGGPGAGGFGAQAPKGGSGSGPSIEDGD |
| 8. | ADQ27307.1 | >gi\|312064069\|gb\|ADQ27307.1\| heat shock 70 kDa protein 1A [*Bos indicus*]  MAKNMAIGIDLGTTYSCVGVFQHGKVEIIANDQGNRTTPSYVAFTDTERLIGDAAKNQVALNPQNTVFDAKRLIGRKFGDPVVQSDMKHWPFRVINDGDKPKVQVSYKGETKAFYPEEISSMVLTKMKEIAEAYLGHPVTNAVTVPAYFNDSQRQATKDAGVIAGLNVLRIINEPTAAAIAYGLDRTGKGERNVLIFDLGGGTFDVSILTIDDGIFEVKATAGDTHLGGEDFDNRLVNHFVEEFKRKHKKDISQNKRAVRRLRTACERAKRTLSSSTQASLEIDSLFEGIDYTSITRARFEELCSDLFRSTLEPVEKALRDAKLDKAQIHDLVLVGGSTRIPKVQKLLQDFFNGRDLNKSINPDEAVAYGAAVQAAILMGDKSENVQDLLLLDVAPLSLGLETAGGVMTALIKRNSTIPTKQTQIFTTYSDNQPGVLIQVYEGERAMTRDNNLLGRFELSGIPPAPRGVPQIEVTFDIDANGILNVTATDKSTGKANKITITNDKGRLSKEEIERMVQEAEKYKAEDEVQRERVSAKNALESYAFNMKSAVEDEGLKGKISEADKKKVLDKCQEVISWLDANTLAEKDEFEHKRKELEQVCNPIISRLYQGAGGPGAGGFGAQAPKGGSGSGPTIEEVD |
| 9. | ADQ27309.1 | >gi\|312064073\|gb\|ADQ27309.1\| heat shock 70 kDa protein 1A [*Bubalus bubalis*]  MAKNMAIGIDLGTTYSCVGVFQHGKVEIIANDQGNRTTPSYVAFTDTERLIGDAAKNQVALNPQNTVFDAKRLIGRKFGDPVVQSDMKHWPFRVINDGDKPKVQVSYKGETKAFYPEEISSMVLTKMKEIAEAYLGHPVTNAVITVPAYFNDSQRQATKDAGVIAGLNVLRIINEPTAAAIAYGLDRTGKGERNVLIFDLGGGTFDVSILTIDDGIFEVKATAGDTHLGGEDFDNRLVNHFVEEFKRKHKKDISQNKRAVRRLRTACERAKRTLSSSTQASLEIDSLFEGIDFYTSITRARFEELCSDLFRSTLEPVEKALRDAKLDKAQIHDLVLVGGSTRIPKVQKLLQDFFNGRDLNKSINPDEAVAYGAAVQAAILMGDKSENVQDLLLLDVAPLSLGLETAGGVMTALIKRNSTIPTKQTQIFTTYSDNQPGVLIQVYEGERAMTRDNNLLGRFELSGIPPAPRGVPQIEVTFDIDANGILNVTATDKSTGKANKITITNDKGRLSKEEIERMVQEAEKYKAEDEVQRERVSAKNALESYAFNMKSAVEDEGLKGKISEADKKKVLDKCQEVISWLDANTLAEKDEFEHKRKELEQVCNPIISRLYQGAGGPGAGGFGAQAPKGGSGSGPTIEEVD |
| 10. | NP_001254803.1 | >gi\|392583892\|ref\|NP_001254803.1\| heat shock 70 kDa protein 1A/1B [*Ovis aries*]  MAKNMAIGIDLGTTYSCVGVFQHGKVEIIANDQGNRTTPSYVAFTDTERLIGDAAKNQVALNPQNTVFDAKRLIGRKFGDPVVQSDMKHWPFRVINDGDKPKVQVSYKGETKAFYPEEISSMVLTKMKEIAEAYLGHPVTNAVITVPAYFNDSQRQATKDAGVIAGLNVLRIINEPTAAAIAYGLHRTGKGERNVLIFDLGGGTFDVSILTIDDGIFEVKATAGDTHLGGEDFDNRLVNHFVEEFKRKHKKDISQNKRAVRRLRTACERAKRTLSSSTQASLEIDSLFEGIDFYTSITRARFEELCSDLFRSTLEPVEKALRDAKLDKAQIHDLVLVGGSTRIPKVQKLLQDFFNGRDLNKSINPDEAVAYGAAVQAAILMGDKSENVQDLLLLDVAPLSLGLETAGGVMTALIKRNSTIPTKQTQIFTTYSDNQPGVLIQVYEGERAMTRDNNLLGRFELSGIPPAPRGVPQIEVTFDIDANGILNVTATDKSTGKANKITITNDKGRLSKEEIERMVQEAEKYKAEDEVQRERVSAKNALESYAFNMKSAVEDEGLKGKISEADKKKVLDKCQEVISWLDANTLAEKDEFEHKRKELEQVCNPIISRLYQGAGGPGAGGFGAQAPKGGSGSGPTIEEVD |
| 11. | ADQ27308.1 | >gi\|312064071\|gb\|ADQ27308.1\| heat shock 70 kDa protein 1A [*Bubalus bubalis*]  MAKNMAIGIDLGTTYSCVGVFQHGKVEIIANDQGNRTTPSYVAFTDTERLIGDAAKNQVALNPQNTVFDAKRLIGRKFGDPVVQSDMKHWPFRVINDGDKPKVQVSYKGETKAFYPEEISSMWLTKMKEIAEAYLGHPVTNAVITVPAYFNDSQRQATKDAGVIAGLNVLRIINEPTAAAIAYGLDRTGKGERNVLIFDLGGGTFDVSILTIDDGIFEVKATAGDTHLGGEDFDNRLVNHFVEEFKRKHKKDISQNKRAVRRLRTACERAKRTLSSSTQASLEIDSLFEGIDFYTSITRARFEELCSDLFRSTLEPVEKALRDAKLDKAQIHDLVLVGGSTRIPKVQKLLQDFFNGRDLNKSINPDEAVAYGAAVQAAILMGDKSENVQDLLLLDVAPLSLGLETAGGVMTALIKRNSTIPTKQTQIFTTYSDNQPGVLIQVYEGERAMTRDNNLLGRFELSGIPPAPRGVPQIEVTFDIDANGILNVTATDKSTGKANKITITNDKGRLSKEEIERMVQEAEKYKAEDEVQRERVSAKNALESYAFNMKSAVEDEGLKGKISEADKKKVLDKCQEVISWLDANTAEKDEFEHKRKELEQVCNPIISRLYQGAGGPGAGGFGAQAPKGGSGSGPTIEEVD |
| 12. | ADH10237.3 | >gi\|311360935\|gb\|ADH10237.3\| 70kDa heat shock protein 1A [*Bubalus bubalis*]  MAKNMAIGIDLGTTYSCVGVFQHGEVEIIANDQGNRTTPSYVAFTDTERLIGDAAKNQVALNPQNTVFDAKRLIGRKFGDPVVQSGMKHWPFRVINDGDKPKVQVSYKGETKAFYPEEISSMVLTKMKEIAEAYLGHPVTNAVITVPAYFNDSQRQATKDAGVIAGLNVLRIINEPTAAAIAYGLDRTGKGERNVLIFDLGGGTFDVSILTIDDGIFEVKATAGDTHLGGEDFDNRLVNHFVEEFKRKHKKDISQNKRAVRRLRTACERAKRTLSSSTQASLEIDSLFEGIDFYTSITRAQFEELCSDLFRSTLEPVEKALRDAKLDKAQIHDLVLVGGSTRIPKVQKLLQDFFNGRDLNKSINPDEAVAYGAAVQAAILMGDKSENVQDLLLLDVAPLSLGLETAGGVMTALIKRNSTIPTKQTQIFTTYSDNQPGVLIQVYEGERAMTRDNNLLGRFELSGIPPAPRGVPQIEVTFDIDANGILNVTATDKSTGKANKITITNDKGRLSKEEIERMVQEAEKYKAEDEVQRERVSAKNALESYAFNMKSAVEDEGLKGKISEADKKKVLDKCQEVISWLDANTLAEKDEFEHKRKELEQVCNPIISRLYQGAGGPGAGGFGAQAPKGGSGSGPTIEEVD |
| 13. | AGK07300.1 | >gi\|481246998\|gb\|AGK07300.1\| heat shock protein 70 A1B [*Camelus dromedarius*]  MAKNTAIGIDLGTTYSCVGVFQHGKVEIIANDQGNRTTPSYVAFTDTERLIGDAAKNQVALNPQNTVFDAKRLIGRKFGDPVVQSDMKHWPFRVINDGDKPKVQVSYKGETKAFYPEEISSMVLTKMKEIAEAYLGHPVTNAVITVPAYFNDSQRQATKDAGVIAGLNVLRIINEPTAAAIAYGLDRTGKGERNVLIFDLGGGTFDVSILTIDDGIFEVKATAGDTHLGGEDFDNRLVNHFVEEFKRKHKKDISQNKRAVRRLRTACERAKRTLSSSTQASLEIDSLFEGIDFYTSITRARFEELCSDLFRSTLEPVEKALRDAKLDKAQIHDLVLVGGSTRIPKVRKLLQDFFNGRDLNKSINPDEAVAYGAAVQAAILMGDKSENVQDLLLLDVAPLSLGLETAGGVMTALIKRNSTIPTKQTQIFATYSDNQPGVLIQVYEGERAMTRDNNLLGRFELSGIPPAPRGVPQIEVTFDIDANGILNVTATDKSTGKANKITITNDKGRLSKEEIERMVQEAEKYKAEDEVQRERVSAKNALESYAFNMKSAVEDEGLKGKISEADKKKVLDKCQEVISWLDANTLAEKDEFEHKRKELEQVCNPIISGLYQGAGGPGAGGFGAQAPKGGSGSGPTIEEVD |
| 14. | AEE00024.1 | >gi\|332078832\|gb\|AEE00024.1\| heat shock protein 70 1A [*Camelus dromedarius*]  MAKNTAIGIDLGTTYSCVGVFQHGKVEIIANDQGNRTTPSYVAFTDTERLIGDAAKNQVALNPQNTVFDAKRLIGRKFGDPVVQSDMKHWPFRVINDGDKPKVQVSYKGETKAFYPEEISSMVLTKMKEIAEAYLGHPVTNAVITVPAYFNDSQRQATKDAGVIAGLNVLRIINEPTAAAIAYGLDRTGKGERNVLIFDLGGGTFDVSILTIDDGIFEVKATAGDTHLGGEDFDNRLVNHFVEEFKRKHKKDISQNKRAVRRLRTACERAKRTLSSSTQASLEIDSLFEGIDFYTSITRARFEELCSDLFRSTLEPVEKALRDAKLDKAQIHDLVLVGGSTRIPKVQKLLQDFFNGRDLNKSINPDEAVAYGAAVQAAILMGDKSENVQDLLLLDVAPLSLGLETAGGVMTALIKRNSTIPTKQTQIFTTYSDNQPGVLIQVYEGERAMTRDNNLLGRFELSGIPPAPRGVPQIEVTFDIDANGILNVTATDKSTGKANKITITNDKGRLSKEEIERMVQEAEKYKAEDEVQRERVSAKNALESYAFNMKSAVEDEGLKGKISEADKKKVLDKCQEVISWLDANTLAEKDEFEHKRKELEQVCNPIISGLYQGAGGPGAGGFGAQAPKGGSGSGPTIEEVD |
| 15. | CAN13333.1 | >gi\|147225156\|emb\|CAN13333.1\| heat shock 70kDa protein 1A [*Sus scrofa*]  MAKSVAIGIDLGTTYSCVGVFQHGKVEIIANDQGNRTTPSYVAFTDTERLIGDAAKNQVALNPQNTVFDAKRLIGRKFGDPVVQADMKHWPFRVINDGDKPKVQVSYKGETKAFYPEEISSMVLTKMKEIAEAYLGHPVSNAVITVPAYFNDSQRQATKDAGVIAGLNVLRIINEPTAAAIAYGLDRTGKGERNVLIFDLGGGTFDVSILTIDDGIFEVKATAGDTHLGGEDFDNRLVNHFVEEFKRKHKKDISQNKRAVRRLRTACERAKRTLSSSTQASLEIDSLFEGIDFYTSITRARFEELCSDLFRSTLEPVEKALRDAKLDKAQIHDLVLVGGSTRIPKVQKLLQDFFNGRDLNKSINPDEAVAYGAAVQAAILMGDKSENVQDLLLLDVAPLSLGLETAGGVMTALIKRNSTIPTKQTQIFTTYSDNQPGVLIQVYEGERAMTRDNNLLGRFELSGIPPAPRGVPQIEVTFDIDANGILNVTATDKSTGKANKITITNDKGRLSKEEIERMVQEAEKYKAEDEIQRERVSAKNALESYAFNMKSAVEDEGLKGKISEADKKKVLDKCQEVISWLDANTLAEKDEFEHKRKELEQVCNPIISGLYQGAGGPGAGGFGAQAPKGGSGSGPTIEEVD |
| 16. | NP_001243852.1 | >gi\|379642967\|ref\|NP_001243852.1\| heat shock 70kDa protein 1A [*Equus caballus*]  MAKSTAIGIDLGTTYSCVGVFQHGKVEIIANDQGNRTTPSYVAFTDTERLIGDAAKNQVALNPQNTVFDAKRLIGRKFGDPVVQSDMKHWPFRVINDGDKPKVQVSYKGETKAFYPEEISSMVLTKMKEIAEAYLGYPVTNAVITVPAYFNDSQRQATKDAGVIAGLNVLRIINEPTAAAIAYGLDRTGKGERNVLIFDLGGGTFDVSILTIDDGIFEVKATAGDTHLGGEDFDNRLVNHFVEEFKRKHKKDISQNKRAVRRLRTACERAKRTLSSSTQASLEIDSLFEGIDFYTSITRARFEELCSDLFRSTLEPVEKALRDAKLDKAQIHDLVLVGGSTRIPKVQKLLQDFFNGRDLNKSINPDEAVAYGAAVQAAILMGDKSENVQDLLLLDVAPLSLGLETAGGVMTALIKRNSTIPTKQTQIFTTYSDNQPGVLIQVYEGERAMTRDNNLLGRFELSGIPPAPRGVPQIEVTFDIDANGILNVTATDKSTGKANKITITNDKGRLSKEEIERMVQEAEKYKAEDEVQRERVSAKNALESYAFNMKSAVEDEGLKGKISEADKKKVLDKCQEVISWLDANTLAEKDEFEHKRKELEQVCNPIITGLYQGAGGPGAGGFGAQAPKGGSGSGPTIEEVD |
| 17. | EAX03528.1 | >gi\|119623933\|gb\|EAX03528.1\| heat shock 70kDa protein 1A [*Homo sapiens*]  MAKAAAIGIDLGTTYSCVGVFQHGKVEIIANDQGNRTTPSYVAFTDTERLIGDAAKNQVALNPQNTVFDAKRLIGRKFGDPVVQSDMKHWPFQVINDGDKPKVQVSYKGETKAFYPEEISSMVLTKMKEIAEAYLGYPVTNAVITVPAYFNDSQRQATKDAGVIAGLNVLRIINEPTAAAIAYGLDRTGKGERNVLIFDLGGGTFDVSILTIDDGIFEVKATAGDTHLGGEDFDNRLVNHFVEEFKRKHKKDISQNKRAVRRLRTACERAKRTLSSSTQASLEIDSLFEGIDFYTSITRARFEELCSDLFRSTLEPVEKALRDAKLDKAQIHDLVLVGGSTRIPKVQKLLQDFFNGRDLNKSINPDEAVAYGAAVQAAILMGDKSENVQDLLLLDVAPLSLGLETAGGVMTALIKRNSTIPTKQTQIFTTYSDNQPGVLIQVYEGERAMTKDNNLLGRFELSGIPPAPRGVPQIEVTFDIDANGILNVTATDKSTGKANKITITNDKGRLSKEEIERMVQEAEKYKAEDEVQRERVSAKNALESYAFNMKSAVEDEGLKGKISEADKKKVLDKCQEVISWLDANTLAEKDEFEHKRKELEQVCNPIISGLYQGAGGPGPGGFGAQGPKGGSGSGPTIEEVD |
| 18. | NP_005336.3 | >gi\|194248072\|ref\|NP_005336.3\| heat shock 70 kDa protein 1A/1B [*Homo sapiens*]  MAKAAAIGIDLGTTYSCVGVFQHGKVEIIANDQGNRTTPSYVAFTDTERLIGDAAKNQVALNPQNTVFDAKRLIGRKFGDPVVQSDMKHWPFQVINDGDKPKVQVSYKGETKAFYPEEISSMVLTKMKEIAEAYLGYPVTNAVITVPAYFNDSQRQATKDAGVIAGLNVLRIINEPTAAAIAYGLDRTGKGERNVLIFDLGGGTFDVSILTIDDGIFEVKATAGDTHLGGEDFDNRLVNHFVEEFKRKHKKDISQNKRAVRRLRTACERAKRTLSSSTQASLEIDSLFEGIDFYTSITRARFEELCSDLFRSTLEPVEKALRDAKLDKAQIHDLVLVGGSTRIPKVQKLLQDFFNGRDLNKSINPDEAVAYGAAVQAAILMGDKSENVQDLLLLDVAPLSLGLETAGGVMTALIKRNSTIPTKQTQIFTTYSDNQPGVLIQVYEGERAMTKDNNLLGRFELSGIPPAPRGVPQIEVTFDIDANGILNVTATDKSTGKANKITITNDKGRLSKEEIERMVQEAEKYKAEDEVQRERVSAKNALESYAFNMKSAVEDEGLKGKISEADKKKVLDKCQEVISWLDANTLAEKDEFEHKRKELEQVCNPIISGLYQGAGGPGPGGFGAQGPKGGSGSGPTIEEVD |
| 19. | ABD96830.1 | >gi\|90657252\|gb\|ABD96830.1\| heat shock 70kDa protein 1A [*Homo sapiens*]  MAKAAAIGIDLGTTYSCVGVFQHGKVEIIANDQGNRTTPSYVAFTDTERLIGDAAKNQVALNPQNTVFDAKRLIGRKFGDPVVQSDMKHWPFQVINDGDKPKVQVSYKGETKAFYPEEISSMVLTKMKEIAEAYLGYPVTNAVITVPAYFNDSQRQATKDAGVIAGLNVLRIINEPTAAAIAYGLDRTGKGERNVLIFDLGGGTFDVSILTIDDGIFEVKATAGDTHLGGEDFDNRLVNHFVEEFKRKHKKDISQNKRAVRRLRTACERAKRTLSSSTQASLEIDSLFEGIDFYTSITRARFEELCSDLFRSTLEPVEKALRDAKLDKAQIHDLVLVGGSTRIPKVQKLLQDFFNGRDLNKSINPDEAVAYGAAVQAAILMGDKSENVQDLLLLDVAPLSLGLETAGGVMTALIKRNSTIPTKQTQIFTTYSDNQPGVLIQVYEGERAMTKDNNLLGRFELSGIPPAPRGVPQIEVTFDIDANGILNVTATDKSTGKANKITITNDKGRLSKEEIERMVQEAEKYKAEDEVQRERVSAKNALESYAFNMKSAVEDEGLKGKISEADKKKVLDKCQEVISWLDANTLAEKDEFEHKRKELEQVCNPIISGLYQGAGGPGPGGFGAQGPKGGSGSGPTIEEVD |
| 20. | AAH02453.1 | >gi\|12803275\|gb\|AAH02453.1\| Heat shock 70kDa protein 1A [*Homo sapiens*]  MAKAAAIGIDLGTTYSCVGVFQHGKVEIIANDQGNRTTPSYVAFTDTERLIGDAAKNQVALNPQNTVFDAKRLIGRKFGDPVVQSDMKHWPFQVINDGDKPKVQVSYKGETKAFYPEEISSMVLTKMKEIAEAYLGYPVTNAVITVPAYFNDSQRQATKDAGVIAGLNVLRIINEPTAAAIAYGLDRTGKGERNVLIFDLGGGTFDVSILTIDDGIFEVKATAGDTHLGGEDFDNRLVNHFVEEFKRKHKKDISQNKRAVRRLRTACERAKRTLSSSTQASLEIDSLFEGIDFYTSITRARFEELCSDLFRSTLEPVEKALRDAKLDKAQIHDLVLVGGSTRIPKVQKLLQDFFNGRDLNKSINPDEAVAYGAAVQAAILMGDKSENVQDLLLLDVAPLSLGLETAGGVMTALIKRNSTIPTKQTQIFTTYSDNQPGVLIQVYEGERAMTKDNNLLGRFELSGIPPAPRGVPQIEVTFDIDANGILNVTATDKSTGKANKITITNDKGRLSKEEIERMVQEAEKYKAEDEVQRERVSAKNALESYAFNMKSAVEDEGLKGKISEADKKKVLDKCQEVISWLDANTLAEKDEFEHKRKELEQVCNPIISGLYQGAGGPGPGGFGAQGPKGGSGSGPTIEEVD |
| 21. | NP_001125893.1 | >gi\|197098904\|ref\|NP_001125893.1\| heat shock 70 kDa protein 1 [*Pongo abelii*]  MAKAAAIGIDLGTTYSCVGVFQHGKVEIIANDQGNRTTPSYVAFTDTERLIGDAAKNQVALNPQNTVFDAKRLIGRKFGDPVVQSDMKHWPFQVNNDGDKPKVQVSYKGETKAFYPEEISSMVLTKMKEIAEAYLGYPVTNAVITVPAYFNDSQRQATKDAGVIAGLNVLRIINEPTAAAIAYGLDRTGKGERNVLIFDLGGGTFDVSILTIDDGIFEVKATAGDTHLGGEDFDNRLVNHFVEEFKRKHKKDISQNKRAVRRLRTACERAKRTLSSSTQASLEIDSLFEGIDFYTSITRARFEELCSDLFRSTLEPVEKALRDAKLDKAQIHDLVLVGGSTRIPKVQKLLQDFFNGRDLNKSINPDEAVAYGAAVQAAILMGDKSENVQDLLLLDVAPLSLGLETAGGVMTALIKRNSTIPTKQTQIFTTYSDNQPGVLIQVYEGERAMTKDNNLLGRFEPSGIPPAPRGVPQIEVTFDIDANGILNVTATDKSTGKANKITITNDKGRLSKEEIERMVQEAEKYKAEDEVQRERVSAKNALESYAFNMKSAVEDEGLKGKISEADKKKVLDKCQEVISWLDANTLAEKDEFEHKRKELEQVCNPIISGLYQGAGGPGPGGFGAQGPKGGSGSGPTIEEVD |
| 22. | CAE83978.1 | >gi\|46237600\|emb\|CAE83978.1\| heat shock 70kD protein 1A [*Rattus norvegicus*]  MAKKTAIGIDLGTTYSCVGVFQHGKVEIIANDQGNRTTPSYVAFTDTERLIGDAAKNQVALNPQNTVFDAKRLIGRKFGDPVVQSDMKHWPFQVVNDGDKPKVQVNYKGENRSFYPEEISSMVLTKMKEIAEAYLGHPVTNAVITVPAYFNDSQRQATKDAGVIAGLNVLRIINEPTAAAIAYGLDRTGKGERNVLIFDLGGGTFDVSILTIDDGIFEVKATAGDTHLGGEDFDNRLVSHFVEEFKRKHKKDISQNKRAVRRLRTACERAKRTLSSSTQASLEIDSLFEGIDFYTSITRARFEELCSDLFRGTLEPVEKALRDAKLDKAQIHDLVLVGGSTRIPKVQKLLQDFFNGRDLNKSINPDEAVAYGAAVQAAILMGDKSENVQDLLLLDVAPLSLGLETAGGVMTALIKRNSTIPTKQTQTFTTYSDNQPGVLIQVYEGERAMTRDNNLLGRFELSGIPPAPRGVPQIEVTFDIDANGILNVTATDKSTGKANKITITNDKGRLSKEEIERMVQEAERYKAEDEVQRERVAAKNALESYAFNMKSAVEDEGLKGKISEADKKKVLDKCQEVISWLDSNTLAEKEEFVHKREELERVCNPIISGLYQGAGAPGAGGFGAQAPKGGSGSGPTIEEVD |
| 23. | NP_114177.2 | >gi\|260064045\|ref\|NP_114177.2\| heat shock 70 kDa protein 1A/1B [*Rattus norvegicus*]  MAKKTAIGIDLGTTYSCVGVFQHGKVEIIANDQGNRTTPSYVAFTDTERLIGDAAKNQVALNPQNTVFDAKRLIGRKFGDPVVQSDMKHWPFQVVNDGDKPKVQVNYKGENRSFYPEEISSMVLTKMKEIAEAYLGHPVTNATVPAYFNDSQRQATKDAGVIAGLNVLRIINEPTAAAIAYGLDRTGKGERNVLIFDLGGGTFDVSILTIDDGIFEVKATAGDTHLGGEDFDNRLVSHFVEEFKRKHKKDISQNKRAVRRLRTACERAKRTLSSSTQASLEIDSLFEGIDFYTSITRARFEELCSDLFRGTLEPVEKALRDAKLDKAQIHDLVLVGGSTRIPKVQKLLQDFFNGRDLNKSINPDEAVAYGAAVQAAILMGDKSENVQDLLLLDVAPLSLGLETAGGVMTALIKRNSTIPTKQTQTFTTYSDNQPGVLIQVYEGERAMTRDNNLLGRFELSGIPPAPRGVPQIEVTFDIDANGILNVTATDKSTGKANKITITNDKGRLSKEEIERMVQEAERYKAEDEVQRERVAAKNALESYAFNMKSAVEDEGLKGKISEADKKKVLDKCQEVISWLDSNTLAEKEEFVHKREELERVCNPIISGLYQGAGAPGAGGFGAQAPKGGSGSGPTIEEVD |
| 24. | AAH54782.1 | >gi\|32451998\|gb\|AAH54782.1\| Heat shock protein 1A [*Mus musculus*]  MAKNTAIGIDLGTTYSCVGVFQHGKVEIIANDQGNRTTPSYVAFTDTERLIGDAAKNQVALNPQNTVFDAKRLIGRKFGDAVVQSDMKHWPFQVVNDGDKPKVQVNYKGESRSFFPEEISSMVLTKMKEIAEAYLGHPVTNAVITVPAYFNDSQRQATKDAGVIAGLNVLRIINEPTAAAIAYGLDRTGKGERNVLIFDLGGGTFDVSILTIDDGIFEVKATAGDTHLGGEDFDNRLVSHFVEEFKRKHKKDISQNKRAVRRLRTACERAKRTLSSSTQASLEIDSLFEGIDFYTSITRARFEELCSDLFRGTLEPVEKALRDAKMDKAQIHDLVLVGGSTRIPKVQKLLQDFFNGRDLNKSINPDEAVAYGAAVQAAILMGDKSENVQDLLLLDVAPLSLGLETAGGVMTALIKRNSTIPTKQTQTFTTYSDNQPGVLIQVYEGERAMTRDNNLLGRFELSGIPPAPRGVPQIEVTFDIDANGILNVTATDKTTGKANKITITNDKGRLSKEEIERMVQEAERYKAEDEVQRDRVAAKNALESYAFNMKSAVEDEGLKGKLSEADKKKVLDKCQEVISWLDSNTLADKEEFVHKREELERVCSPIISGLYQGAGAPGAGGFGAQAPKGASGSGPTIEEVD |
| 25. | NP_034609.2 | >gi\|124339829\|ref\|NP_034609.2\| heat shock 70 kDa protein 1A [*Mus musculus*]  MAKNTAIGIDLGTTYSCVGVFQHGKVEIIANDQGNRTTPSYVAFTDTERLIGDAAKNQVALNPQNTVFDAKRLIGRKFGDAVVQSDMKHWPFQVVNDGDKPKVQVNYKGESRSFFPEEISSMVLTKMKEIAEAYLGHPVTNAVITVPAYFNDSQRQATKDAGVIAGLNVLRIINEPTAAAIAYGLDRTGKGERNVLIFDLGGGTFDVSILTIDDGIFEVKATAGDTHLGGEDFDNRLVSHFVEEFKRKHKKDISQNKRAVRRLRTACERAKRTLSSSTQASLEIDSLFEGIDFYTSITRARFEELCSDLFRGTLEPVEKALRDAKMDKAQIHDLVLVGGSTRIPKVQKLLQDFFNGRDLNKSINPDAVAYGAAVQAAILMGDKSENVQDLLLLDVAPLSLGLETAGGVMTALIKRNSTIPTKQTQTFTTYSDNQPGVLIQVYEGERAMTRDNNLLGRFELSGIPPAPRGVPQIEVTFDIDANGILNVTATDKSTGKANKITITNDKGRLSKEEIERMVQEAERYKAEDEVQRDRVAAKNALESYAFNMKSAVEDEGLKGKLSEADKKKVLDKCQEVISWLDSNTLADKEEFVHKREELERVCSPIISGLYQGAGAPGAGGFGAQAPKGASGSGPTIEEVD |
| 26. | EGV95270.1 | >gi\|344239167\|gb\|EGV95270.1\| Heat shock 70 kDa protein 1A [*Cricetulus griseus*]  MAKSTAIGIDLGTTYSCVGVFQHGKVEIIANDQGNRTTPSYVAFTDTERLIGDAAKNQVALNPQNTVFDAKRLIGRKFGDAVVQADMKHWPFQVVNDGDKPKVQVSYKGETRAFYPEEISSMVLTKMKEVAEAYLGHPVTNAVITVPAYFNDSQRQATKDAGVIAGLNVLRIINEPTAAAIAYGLDRSGKGERNVLIFDLGGGTFDVSILTIDDGIFEVKATAGDTHLGGEDFDNRLVSHFVEEFKRKHKKDISQNKRAVRRLRTACERAKRTLSSSTQANLEIDSLFEGIDFYTSITRARFEELCADLFRGTLEPVEKSLRDAKMDKAKIHDIVLVGGSTRIPKVQKLLQDFFNGRDLNKSINPDEAVAYGAAVQAAILMGDKSENVQDLLLLDVAPLSLGLETAGGVMTALIKRNSTIPTKQTQTFTTYSDNQPGVLIQVYEGERAMTRDNNLLGRFELSGIPPAPRGVPQIEVTFDIDANGILNVTATDKSTGKANKITITNDKGRLSKEEIERMVQEAERYKAEDEVQRERVAAKNALESYAFNMKSAVEDEGLKGKISEADRKKVLDGCQEVISWLDANTLADKEEFVHKRQELERVCGPIVSGLYQGAGAPGAGGFGAQAPKGGSGSGPTIEEVD |
| 27. | ABS44989.1 | >gi\|152941104\|gb\|ABS44989.1\| heat shock 70kDa protein 1A [*Bos taurus*]  MSARGPAIGIDLGTTYSCVGVFQHGKVEIIANDQGNRTTPSYVAFTDTERLIGDAAKNQVAMNPTNTIFDAKRLIGRKFEDATVQSDMKHWPFRVVSEGGKPKVQVEYKGEIKTFFPEEISSMVLTKMKEIAEAYLGGKVQSAVITVPAYFNDSQRQATKDAGTITGLNVLRIINEPTAAAIAYGLDKKGCAGGEKNVLIFDLGGGTFDVSILTIEDGIFEVKSTAGDTHLGGEDFDNRMVSHLAEEFKRKHKKDIAPNKRAVRRLRTACERAKRTLSSSTQASIEIDSLYEGVDFYTSITRARFEELNADLFRGTLEPVEKALRDAKLDKGQIQEIVLVGGSTRIPKIQKLLQDFFNGKELNKSINPDEAVAYGAAVQAAILIGDKSENVQDLLLLDVTPLSLGIETAGGVMTPLIKRNTTIPTKQTQTFTTYSDNQSSVLVQVYEGERAMTKDNNLLGKFDLTGIPPAPRGVPQIEVTFDIDANGILNVTAADKSTGKENKITITNDKGRLSKDDIDRMVQEAERYKSEDEANRDRVAAKNAVESYTYNIKQTVEDEKLRGKISDQDKNKILDKCQEVINWLDRNQMAEKDEYEHKQKELERVCNPIISKLYQGGPGGGGGSGASGGPTIEEVD |
| 28. | NP_001167480.1 | >gi\|291290899\|ref\|NP_001167480.1\| heat shock 70kDa protein 1A [*Xenopus laevis*]  MSKGPAVGIDLGTTYSCVGVFQHGKVEIIANDQGNRTTPSYVAFTDTERLIGDAAKNQVAMNPTNTVFDAKRLIGRRFDDPVVHSDMKHWPFNVINDGGRPKVQVEYKGETKTFYAEEVSSMVLIKMKEIAEAYLGKTITNAVITVPAYFNDSQRQATKDAGTISGLNVLRIINEPTAAAIAYGLDKKVGSERNVLIFDLGGGTFDVSILTIEDGIFEVKSTAGDTHLGGEDFDNRMVNHFVGEFKRKHKKDITENKRAVRRLRTACERAKRTLSSSTQASIEIDSLYEGIDFYTSLTRARFEELNADLFRGTLEPVEKALRDAKMDKAQIHDIVLVGGSTRIPKIQKLLQDFFNGRELNKSINPDEAVAYGAAVQAAILSGDKSENVQDLLLLDVTPLSLGIETAGGVMTVLIKRNTTIPTKQTQTFTTYSDNQPGVLIQVFEGERAMTKDNNLLGKFELTGIPPAPRGVPQIEVTFDIDANGIMNVSAVDKSTGKENKITITNDKGRLSKDEIERMVQEADRYKTEDEAQRDKISAKNSLESLAFNMKSTVEDEKLKDKISQEDKQKILDKCNEVISWLDRNQMAEKEEYEHQQKELQNLCNPIITKLYQGAGGAGMPGGMPGGMPGGFPGAGAGAGGGGSSGPTIEEVD |
| 29. | AGO01980.1 | >gi\|512393022\|gb\|AGO01980.1\| heat shock 70kDa protein 1a [*Monopterus albus*]  MSAAKGVAIGINLGTTYSCVGVFQHGKVEIIANDQGNRTTPSYVAFTDTERLIGDAAKNQVALNPSNTVFDAKRLIGRKFDDPVVQADMKHWPFKVLSDGGKPKIQVEYKGENKAFYPEEISSMVLVKMKEIAEAYLGHKVSNAVITVPAYFNDSQRQATKDAGVIAGLNVLRIINEPTAAAIAYGLDKGKTGERNVLIFDLGGGTFDVSILTIEDGIFEVKATAGDTHLGGEDFDNRMVNHFVEEFKRKHKKDISQNKRALRRLRTACERAKRTLSSSSQASIEIDSLYEGIDFYTSITRARFEELCSDLFRGTLEPVEKALRDAKMDKAQIHDVVLVGGSTRIPKIQKLLQDFFNGRELNKSINPDEAVAYGRAVQAAILTGDTSGNVQDLLLLDVAPLSLGIETAGGVMTSLIKRNTTIPTKQTQVFTTYSDNQPGVLIQVYEGERAMTKDNNLLGKFELAGIPPAPRGVPQIEVTFDIDANGILNVSAVDKSTGQENKITITNDKGRLSKEEIERMVQDADKYKAEDDIQREKIAAKNSLESYAFNVKSSVQDEKLRDKMSEEDRKKVIEKCDEAIMWLENNQLAEKEEYQHKLKELENVCNPIISKLYQGGMPTGSTCGQQARAGAEGPTIEEVD |
| 30. | CDI70365.1 | >gi\|553727404\|emb\|CDI70365.1\| heat shock 70 kDa protein 1a [*Echinococcus multilocularis*]  MSARPAIGIDLGTTFSCVGVFQSGRVEIIANDQGNRITPSCVAFTDEERLIGESAINQAAMNVANTVLDVKRLIGRRFDDEVVQSDMKHWPFKVINSEGKPKIEVQHCGKTKQFVAEQISAMVLSKMKETAEAYLGEEVTDAVVTVPAYFNDNQRNATIDAAKIAGLNVLRLVNEPTAAAIAYGMDRTSDRQRNVLIFDWGGGTFDVSILSIKNREFEVKAVGGDTHLGGEDITSRLVDHFVEVIKHEHRGKDLTTNKRAIHRLRKACETAKRMLSAAASTKIAVESLFDEVDFCKTLARAEFEQLCMDLFSQTMDKVETTLSDAKLRKADIHEILLVGGSTRIPKVQSMLQDFFNGRDLQRSIPDEAVAYGAAVLAAKLSGNMSKLMENLMLYEVTPLSLGWKDCYGAMVTVIKRNTRIPTKRTCSSQTASDNQTSVRTSIFEGERAMAKDNNFLGEFLVQGFPAKPRGEIKFEDTFEIDENGILHVSSVEKSTGKQNSITITKYKGHLSEEEAKLMLAEAEKFKQEDEKERSRVAAMNALLDCIYSTKRKLEKVEVKQKMSEKNRKMLLAKCEESIKWADREKQATKEDYERKRKQFESECSL |
| 31. | ADQ27306.1 | >gi\|312064067\|gb\|ADQ27306.1\| heat shock 70 kDa protein 1A [*Bos indicus*]  MAKNMAIGIDLGTTYSCVGVFQHGKVEIIANDQGNRTTPSYVAFTDTERLIGDAAKNQVALNPQNTVFDAKRLIGRKFGDPVVQSDMKHWPFRVINDGDKPKVQVSYKGETKAFYPEEISSMVLTKMKEIAEAYLGHPVTNAVITVPAYFNDSQRQATKDAGVIAGLNVLRIINEPTAAAIAYGLDRTGKGERNVLIFDLGGGTFDVSILTIDDGIFEVKATAGDTHLGGEDFDNRLVNHFVEEFKRKHKKDISQNKRAVRRLRTACERAKRTLSSSTQASLEIDSLFEGIYFYTSITRAWFEELCSDLFRSTLEPVEKALRDAQLDKAQIHDLVLVGGSTRIPKVQKLLQDFFNGRDLNKSINPDEAVAYGAAVQAAILMGDKSENVQDLLLLDVAPLSLGLETAGGVMTALIKRNSTIPTKQTQIFTTYSDNQPGVLIQVYEGERAMTRDNNLLGRFELSGIPPAPRGVPQIEVTFDIDANGILNVTATDKSTGKANKITITNDKGRLSKEEIERMVQEAEKYKAEDEVQRERVSAKNALESYAFNMKSAVEDEGLKGKISEADKKKVLDKCQEVISWLDANTLAEKDEFEHKRKELEQVCNPIISRLYQGAGGPGAGGFGAQGPKGGSGSGPTIEEVD |
| 32. | AAN78092.1 | >gi\|32364477\|gb\|AAN78092.1\| heat-shock 70- kDa protein 1A [*Bos taurus*]  MAKNMAIGIDLGTTYSCVGVFQHGKVEIIANDQGNRTTPSYVAFTDTERLIGDAAKNQVALNPQNTVFDAKRLIGRKFGDPVVQSDMKHWPFRVINDGDKPKVQVSYKGETKAFYPEEISSMVLTKMKEIAEAYLGHPVTNAVITVPAYFNDSQRQATKDAGVIAGLNVLRIINEPTAAAIAYGLDRTGKGERNVLIFDLGGGTFDVSILTIDDGIFEVKATAGDTHLGGEDFDNRLVNHFVEEFKRKHKKDISQNKRAVRRLRTACERAKRTLSSSTQASLEIDSLFEGIDFYTSITRARFEELCSDLFRSTLEPVEKALRDAKLDKAQIHDLVLVGGSTRIPKVQKLLQDFFNGRDLNKSINPDEAVAYGAAVQAAILMGDKSENVQDLLLLDVAPLSLGLETAGGVMTALIKRNSTIPTKQTQIFTTYSDNQPGVLIQVYEGERAMTRDNNLLGRFELSGIPPAPRGVPQIEVTFDIDANGILNVTATDKSTGKANKITITNDKGRLSKEEIERMVQEAEKYKAEDEVQRERVSAKNALESYAFNMKSAVEDEGLKGKISEADKKKVLDKCQEVISWLDANTLAEKDEFEHKRKELEQVCNPIISRLYQGAGGPGAGGFGAQGPKGGSGSGPTIEEVD |
| 33. | AAN78094.1 | >gi\|32364480\|gb\|AAN78094.1\| heat-shock 70- kDa protein 1A [*Bos taurus*]  MAKNMAIGIDLGTTYSCVGVFQHGKVEIIANDQGNRTTPSYVAFTDTERLIGDAAKNQVALNPQNTVFDAKRLIGRKFGDPVVQSDMKHWPFRVINDGDKPKVQVSYKGETKAFYPEEISSMVLTKMKEIAEAYLGHPVTNAVITVPAYFNDSQRQATKDAGVIAGLNVLRIINEPTAAAIAYGLDRTGKGERNVLIFDLGGGTFDVSILTIDDGIFEVKATAGDTHLGGEDFDNRLVNHFVEEFKRKHKKDISQNKRAVRRLRTACERAKRTLSSSTQASLEIDSLFEGIDFYTSITRARFEELCSDLFRSTLEPVEKALRDAKLDKAQIHDLVLVGGSTRIPKVQKLLQDFFNGRDLNKSINPDEAVAYGAAVQAAILMGDKSENVQDLLLLDVAPLSLGLETAGGVMTALIKRNSTIPTKQTQIFTTYSDNQPGVLIQVYEGERAMTRDNNLLGRFELSGIPPAPRGVPQIEVTFDIDANGILNVTATDKSTGKANKITITNDKGRLSKEEIERMVQEAEKYKAEDEVQRERVSAKNALESYAFNMKSAVEDEGLKGKISEADKKKVLDKCQEVISWLDANTLAEKDEFEHKRKELEQVCNPIISRLYQGAGGPGAGGFGAQGPKGGSGSGPTIEEVD |
